# Gypmacrophin A, a Rare Pentacyclic Sesterterpenoid, Together with Three Depsides, Functioned as New Chemical Evidence for *Gypsoplaca macrophylla* (Zahlbr.) Timdal Identification

**DOI:** 10.3390/molecules22101675

**Published:** 2017-10-09

**Authors:** Yuan-Fei Zhou, Hai-Xia Shi, Kun Hu, Jian-Wei Tang, Xing-Ren Li, Xue Du, Han-Dong Sun, Li-Song Wang, Jian-Xin Pu

**Affiliations:** 1 State Key Laboratory of Phytochemistry and Plant Resources in West China, Kunming Institute of Botany, Chinese Academy of Sciences, Kunming 650201, China; zhouyuanfei@mail.kib.ac.cn (Y.-F.Z.); hukun@mail.kib.ac.cn (K.H.); tangjianwei@mail.kib.ac.cn (J.-W.T.); lixingren@mail.kib.ac.cn (X.-R.L.); duxue@mail.kib.ac.cn (X.D.); hdsun@mail.kib.ac.cn (H.-D.S.); 2 Kunming College of Life Sciences, University of Chinese Academy of Sciences, Beijing 100039, China; 3 Key Laboratory for Plant Biodiversity and Biogeography of East Asia, Kunming Institute of Botany, Chinese Academy of Sciences, Kunming 650201, China; shihaixia@mail.kib.ac.cn

**Keywords:** Gypsoplacaceae, *Gypsoplaca macrophylla*, sesterterpenoid, identification

## Abstract

The phytochemical investigation on 1 g of materials from *Gypsoplaca macrophylla* (Zahlbr.) Timdal resulted in the discovery of gypmacrophin A, a rare pentacyclic sesterterpenoid; brialmontin III, a new polysubstituted depside and two known ones, brialmontins I and II. The structure and absolute configurations of gypmacrophin A were elucidated by spectroscopic analyses and computational methods. Gypmacrophin A showed weak inhibition of AchE with an IC_50_ value of 32.03 μM. The four compounds provided new chemical evidence for *G. macrophylla* identification.

## 1. Introduction

Sesterterpenoids, a class of terpenoids assembled from five isoprene units, are a relatively small group of terpenoids distributed in plants, lichens, endophytic fungi, and marine organisms. These molecules were found to exhibit versatile biological activities, such as anti-inflammatory [1], anticancer [2], antimicrobial [3], antitubercular [4], and anti-biofilm activities [5]. Furthermore, the structural complexity of sesterterpenoids have made them attractive to synthetic and biomedical purposes [6]. Among the previously reported sesterterpenoids, those with pentacyclic carbon scaffold were the rarest, yet possessed the most complicated architectures [7,8], including those originating from gene mining and heterologous expression [9,10].

**Figure 1:**
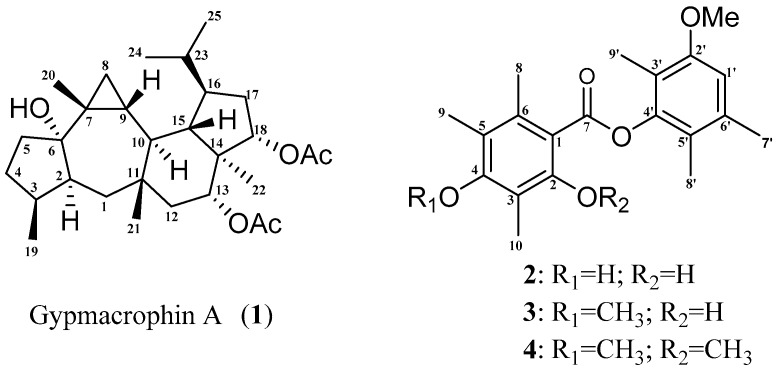
Structure of gypmacrophin A (**1**) and brialmontins I-III (**2**–**4**).

Lichens are symbiotic organisms resulting from an association between mycobionts (fungus) and photobionts (green alga or cyanobacteria) [11], which led to production of a large quantity of fascinating secondary metabolites. Lichens in one genus or family often share similar specific metabolites. Secondary metabolites in lichens carry valuable information on phylogenetic relationships [12] that they are often reported along with internal transcribed spacer (ITS) sequences when publishing new lichen species [13,14]. As for *Gypsoplaca macrophylla*, a single species in the genus *Gypsoplaca* and *Gypsoplacaceae* family, no distinctive components have been reported, including the claimed triterpenoids [15], since the species was first published in 1930 [16]. Hence, there was no valuable chemical evidence to help lichenologists to authenticate them. The present investigation on 1 g of materials from *G. macrophylla* resulted in the discovery of gypmacrophin A, a rare pentacyclic sesterterpenoid; brialmontin III, a new polysubstituted depside, and two known depsides, brialmontins I and II (Figure 1). The structure and absolute configurations of gypmacrophin A were elucidated by spectroscopic analyses and computational methods. These four compounds provided new chemical evidence for *G. macrophylla* identification.

## 2. Results and Discussion

Gypmacrophin A (**1**) was obtained as a white amorphous powder, ${\left[\alpha \right]}_{D}^{25}$ +20.8 (*c* 0.2, MeOH). The molecular formula of **1** was established as C_29_H_46_O_5_ from its high resolution electrospray ionization mass spectroscopy (HR-ESI-MS) (*m*/*z* 497.3238 [M + Na]^+^, calcd. for C_29_H_46_NaO_5_: 497.3237), indicating seven degrees of unsaturation. The ^1^H-NMR (Table 1) of **1** exhibited signals for three secondary methyl groups at *δ*_H_ 0.83 (3H, d, *J* = 7.4, Me-19), 0.85 (3H, d, *J* = 7.3, Me-24), and 0.86 (3H, d, *J* = 7.3, Me-25), and resonated five tertiary methyls at *δ*_H_ 0.92 (3H, s, Me-22), 1.03 (3H, s, Me-21), 1.07 (3H, s, Me-20), 1.90 (3H, s, AcO-18), and 1.93 (3H, s, AcO-13). In the lower field region, two signals at *δ*_H_ 4.65 (1H, t, *J* = 9.0 Hz, H-18) and 4.91 (1H, dd, *J* = 11.7, 4.9 Hz, H-13) were ascribed as methines. Twenty-nine carbon signals, including eight methyl, six methylene, nine methine, and six quaternary carbons (including two carbonyl carbons), were evidenced from its ^13^C-NMR (Table 1), DEPT, and HSQC spectra. Since two in seven degrees of unsaturation were occupied by the carbonyl groups, compound **1** was deduced as a sesterterpenoid possessing a pentacyclic ring system.

**Figure 2:**
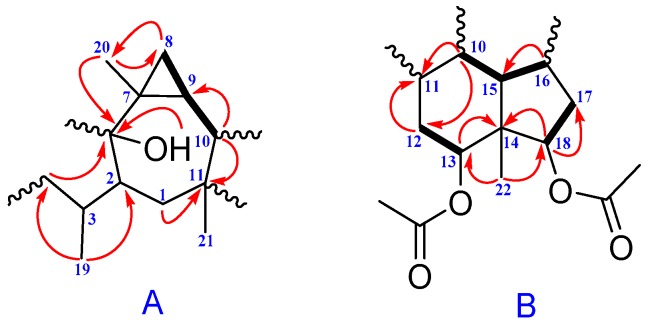
^1^H–^1^H COSY (bold) and selected HMBC (arrow) correlations of **1**.

The gross structure of **1** was determined by 2D-NMR studies. The first part of HMBC correlations from H_3_-19 (*δ*_H_ 0.83, d) to C-2 (*δ*_C_ 49.7), C-3 (*δ*_C_ 37.7) and C-4 (*δ*_C_ 32.4); from H_3_-20 (*δ*_H_ 1.07, s) to C-8 (*δ*_C_ 16.4), C-7 (*δ*_C_ 24.2) and C-6 (*δ*_C_ 83.3); from H-10 (*δ*_H_ 1.76, t) to C-9 (*δ*_C_ 24.8) and C-11; from H-1*β* (*δ*_H_ 1.41, overlapped) to C-2 and C-11; from H_3_-21 (*δ*_H_ 1.03, s) to C-11; from H-4*β* (*δ*_H_ 1.29, m) to C-6 together with ^1^H–^1^H COSY couplings of H-8/H-9/H-10 could establish the fragment A (Figure 2). The mere lone hydroxy group was attached to C-6 according to chemical shift, and the key HMBC correlation with C-6.

**Figure 3:**
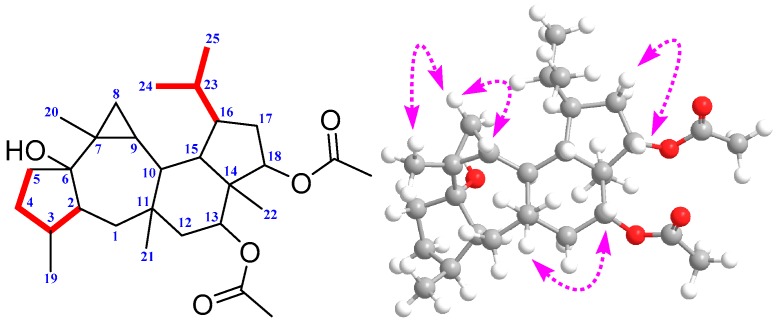
Selected HSQC-TOCSY (red bold) and ROESY correlations of **1**.

The second part HMBC correlations from H_3_-22 (*δ*_H_ 0.92, s) to C-14 (*δ*_C_ 49.4), C-18 (*δ*_C_ 81.1), C-13 (*δ*_C_ 77.3) and C-15 (*δ*_C_ 44.6); from H-16 (*δ*_H_ 2.02, overlapped) to C-17 (*δ*_C_ 28.7); from H-15 (*δ*_H_ 1.60, m) to C-11 (*δ*_C_ 39.7) and C-17; from H-12*α* (*δ*_H_ 1.60 overlapped) to C-11; from H-10 (*δ*_H_ 1.76, t) to C-15, C-14 and C-11 together with ^1^H–^1^H COSY couplings of H-12/H-13, H-10/H-15, H-15/H-16 and H-17/H-18 could establish the fragment B (Figure 2). Two acetoxy groups were observed to attach on C-13 and C-18, according to the chemical shift values and HMBC correlations, respectively. However, the remaining two methyls (C-24, C-25), one methylene (C-5) and one methine (C-23), had no valuable correlations with other atoms from these spectra, which presented us an obstacle in terms of elucidating them clearly. A HSQC-TOCSY experiment was conducted to figure them out. The HSQC-TOCSY correlations of H-4, H-3, and H-2 to C-5 (*δ*_C_ 38.8); H-24 and H-25 to C-23 (*δ*_C_ 28.4) were constructed of the two below parts. Combining fragment A with fragment B according to the ^1^H–^1^H COSY couplings of H-9/H-10 and H-10/H-15, along with HMBC correlation of H-12*α* to C-1, the planar structure of compound **1** was deduced in Figure 3.

The relative stereochemistry of **1** could be elucidated using data obtained from a ROESY experiment. The ROESY spectrum of **1** showed the correlations between the proton of H-2 and H-1*α*, H-2 and H-10, H-10 and H_3_-22, H-17*α* and H-16, together with HO-6 and H-8*α*, suggesting that HO-6, H-2, H-3, H-10, H-16, H_3_-22, and H-8*α* had the same orientation. On the contrary, ROESY correlations between H-8*β* and H_3_-20, H_3_-20 and H-9; H-9 and H_3_-21, H-15 and H-13, H-13 and H-12*β* as well as H-17*β* and H-18 suggested that these protons were located in the same orientation (Figure 3). Therefore, the relative configuration of **1** was determined as 2*S*′, 3*S*′, 6*S*′, 7*R*′, 9*R*′, 10*S*′, 11*S*′, 13*R*′, 14*S*′, 15*S*′, 16*R*′, 18*S*′.

**Figure 4:**
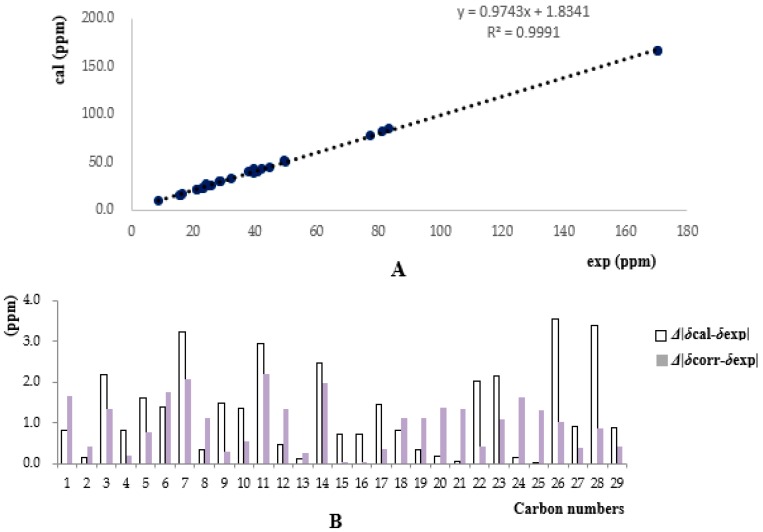
(**A**) Regression analysis of experimental versus calculated ^13^C-NMR chemical shifts of **1** at MPW1PW91/6-31G (d,p) level; linear fitting was shown as a line; (**B**) Absolute and relative chemical shift errors between *δ*cal/*δ*exp (mean absolute error (MAE) = 1.3) and *δ*corr/*δ*exp (corrected mean absolute error (CMAE) = 1.0).

To further confirm the characteristic structure and determine the absolute configuration of **1**, a quantum chemical calculation was resorted to in our elucidation work, after several failures to obtain a single crystal. Overall, conformation searches on **1** gave nine conformers with populations higher than 1%. All of these conformers were optimized at the theory of B3LYP/6-31G (d,p) to give six conformers. These predominant conformers were subjected to NMR calculations at the PW1PW91/6-31G (d,p) level with polarizable continuum (PCM) model in acetone. As shown (Table 1), the calculated ^13^C-NMR of **1** matched well with the experimental one, giving compelling support to verify the aforementioned structure. The correlation coefficient (*R*^2^) (Figure 4) between the experimental and calculated data from linear regression analysis was 0.9991, and the mean absolute error (MAE) and the corrected mean absolute error (CMAE) were 1.3 and 1.0 ppm, respectively, thus supporting the structural rationality of **1**. For absolute configuration, the low cotton effect of electronic circular dichroism forced us to seek help from the optical rotation calculations, which had also been successfully applied in deciding configurations of natural products [17]. Optical rotation calculations of **1** were carried at the B3LYP/6-31+G (d,p) and B3LYP/6-311++G (2d,p) level, with PCM model in MeOH based on B3LYP/6-31G (d,p) optimized geometries. The calculated specific rotation (+83.4°) of (2*S*, 3*S*, 6*S*, 7*R*, 9*R*, 10*S*, 11*S*, 13*R*, 14*S*, 15*S*, 16*R*, 18*S*)-**1** was close to the experimental value (${\left[\alpha \right]}_{D}^{25}$ +20.8, *c* 0.2, MeOH), while the calculated data for its enantiomer had a total opposite result. Therefore, the absolute configuration of **1** was defined to be 2*S*, 3*S*, 6*S*, 7*R*, 9*R*, 10*S*, 11*S*, 13*R*, 14*S*, 15*S*, 16*R*, 18*S*.

**Figure 5:**
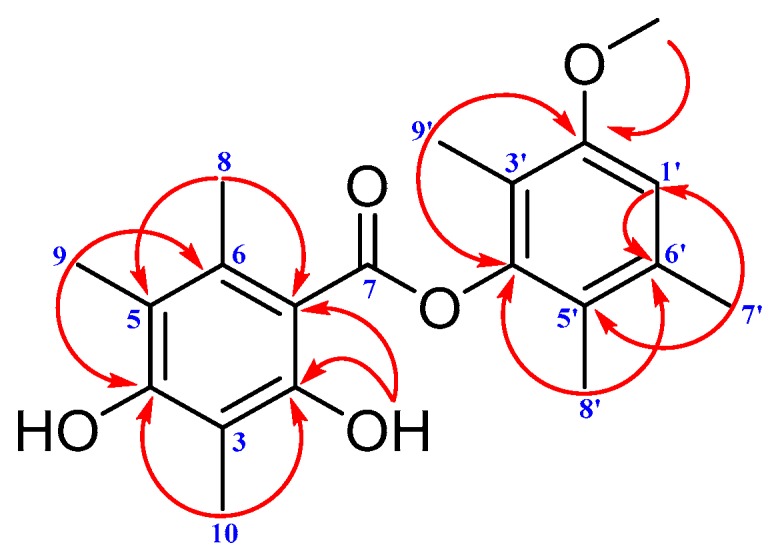
Key HMBC (arrows) correlations of **2**.

Brialmontin III (**2**) was obtained as a white amorphous powder. The molecular formula was determined as C_20_H_24_O_5_ by its HR-ESI-MS peak at *m*/*z* 367.1509 [M + Na]^+^ (calcd. for C_20_H_24_NaO_5_: 367.1516), indicating nine degrees of unsaturation. The ^1^H-NMR spectrum of **2** exhibited signals for seven methyl groups, 1.99 (3H, s, Me-9’), 2.02 (3H, s, Me-8’), 2.16 (3H, s, Me-10), 2.23 (3H, s, Me-9), 2.31 (3H, s, Me-7’), 2.66 (3H, s, Me-8) and 3.85 (3H, s, CH_3_O-2’); one methine proton, 6.79 (1H, s, H-1’); two hydroxy protons, 8.18 (1H, s, HO-4) and 11.56 (1H, s, HO-2). Twenty carbon signals, including seven methyl, one methine, and twelve quaternary carbons (including one carbonyl carbon) were evidenced by the ^13^C-NMR (Table 2) and HSQC spectra. Considering the similar single peak and chemical shift values of **2** with those of **3**, compound **2** was deduced as a depside with polysubstitution of methyl and methoxy groups [18]. The precise structure of **2** was determined by interpretation of 2D-NMR experiments. The HMBC correlations (Figure 5) from H-1’ to C-2’ (*δ*_C_ 156.8) and C-6’ (*δ*_C_ 121.0); protons of methoxy group to C-2’; H_3_-9’ to C-2’, C-3’ (*δ*_C_ 116.6) and C-4’ (*δ*_C_ 149.2); H_3_-8’ to C-4’, C-5’ (*δ*_C_ 136.1), and C-6’ as well as H_3_-7’ to C-1’, C-5’ and C-6’ could establish a phenyl ring. The rest signals of HMBC correlations from H_3_-10 to C-2, C-3 and C-4; proton of another hydroxy group to C-1 (*δ*_C_ 105.1), C-2 (*δ*_C_ 161.8) and C-3 (*δ*_C_ 109.3); H_3_-9 to C-4 (*δ*_C_ 159.8), C-5 (*δ*_C_ 117.3) and C-6 (*δ*_C_ 138.4) together with the correlations from H_3_-8 to C-1, C-5 and C-6 could construct another phenyl ring. The carbonyl group connected the two parts functioned as a bridge, positioning suitable carbons (C-1, C-4’) on the basis of chemical shift.

Compounds **3** (brialmontin I) and **4** (brialmontin II) were confirmed by comparing their chemical shifts data of ^1^H- and ^13^C-NMR with the literature [18,19]. The two ones were confirmed as polysubstituted depsides as well. Interestingly, brialmontin I has only been found in two lichens, *Lecania brialmontii* and *Lecidella carpathica*, while brialmontin II only has been discovered in *L. brialmontii*. It is likely that *G. macrophylla* has similar synthetic gene clusters with these two species above, concerning brialmontins I and II. Therefore, deep research needs to be done to discuss the systematic relationships among these three voucher specimens. Sesterterpenoids bearing such pentacyclic carbon skeletons have only been reported once [20]. Since more biological activities of such unique skeletons need to be discovered, gypmacrophin A was then investigated for its inhibitory activity against AchE (Acetylcholinesterase). Brialmontins I, II, and III were excluded from biological evaluation because of sample shortage. The result showed that gypmacrophin A was a weak inhibitor of AchE with an IC_50_ value of 32.03 μM, with positive control giving an IC_50_ value of 0.24 μM.

## 3. Experimental Section

### 3.1. General Experimental Procedures

Optical rotations were measured with a JASCO P-1020 polarimeter (Jasco, Tokyo, Japan). UV spectra were obtained using a Shimadzu UV-2401 PC spectrophotometer (Shimadzu, Kyoto, Japan). A Tensor 27 spectrophotometer (Bruker, Karlsruhe, Germany) was used for scanning IR spectroscopy with KBr pellets. 1D- and 2D-NMR spectra were recorded on Bruker DRX-600 and DRX-800 spectrometers (Bruker Biospin, Zurich, Switzerland) with TMS as internal standard. Chemical shifts (*δ*) are expressed in parts per million (ppm) with reference to the solvent signals. HR-ESI-MS was performed on an API QSTAR spectrometer (Agilent Technologies, Santa Clara, CA, USA). Semi-preparative HPLC was performed on an Agilent 1200 liquid chromatograph (Agilent Technologies, Santa Clara, CA, USA) with a Zorbax SB-C18 (9.4 mm × 250 mm) column (Agilent Technologies, Santa Clara, CA, USA). Column chromatography (CC) was performed with RP-18 (300–400 mesh, Qingdao Marine Chemical, Inc., Qingdao, China). All solvents used for CC and HPLC were of analytical grade (Shanghai Chemical Reagents Co., Ltd., Shanghai, China) and chromatographic grade (Dikma Technologies Inc., Beijing, China), respectively.

### 3.2. Collection and Identification of Biological Materials

The Chinese Specimens used in the study were collected from Yunnan provinces and were identified by Professor Li-Song Wang of the Kunming Institute of Botany, Chinese Academy of Sciences (CAS). Morphological characters and sequences of ITS and nrLSU were compared with specimens from the Museum of Evolution (UPS, Oslo, Norway) and the Field Museum of Natural History (F, Chicago, IL, USA). A voucher specimen Wang Lisong et al. 15-49586 (KUN-L-52617) was deposited in the herbarium of the Kunming Institute of Botany, Chinese Academy of Sciences (KUN-L, Kunming, China).

### 3.3. Extraction and Isolation

The air-dried lichens, *G. macrophylla* (1 g), were extracted with acetone (20 mL) three times. After removal of acetone under reduced pressure, the residue (34 mg) was subjected to RP-18 eluted with a MeOH–H_2_O gradient (30:70–100:0) to yield A–D fractions. Fraction D (11 mg) was further chromatographed on semi-preparative semi-preparative high performance liquid chromatography (HPLC) with CH_3_CN–H_2_O (90:10) to afford two new compounds, named as gypmacrophin A (**1**) (3.8 mg) and brialmontin III (**2**) (0.5 mg), and two known ones, brialmontins I (**3**) (0.5 mg) and II (**4**) (0.8 mg).

Gypmacrophin A (**1**) was obtained as a white amorphous powder: ${\left[\alpha \right]}_{D}^{25}$ +20.8 (*c* 0.2, MeOH); UV (MeOH) *λ*_max_ (log*ε*): 203 (3.30), 210 (3.25) nm; IR (KBr) *v*_max_: 3440, 2956, 1739 cm^−1^. ^1^H- and ^13^C-NMR data: (Table 1); (+)-HR-ESI-MS *m*/*z* 497.3238 [M + Na]^+^ (calcd. for C_29_H_46_NaO_5_: 497.3237).

Brialmontin III (**2**) was obtained as a white amorphous powder: UV (MeOH) *λ*_max_ (log*ε*): 202 (4.91), 216 (4.87), 262 (4.37), 323 (3.84) nm; IR (KBr) *v*_max_: 3447, 2917, 1634 cm^−1^. ^1^H- and ^13^C-NMR data: (Table 2); (+)-HR-ESI-MS *m*/*z* 367.1509 [M + Na]^+^ (calcd. for C_20_H_24_NaO_5_: 367.1516).

Brialmontin I (**3**) was obtained as a white amorphous powder: ^1^H- and ^13^C-NMR data: (Table 2); (+)-ESI-MS *m*/*z* 381 [M + Na]^+^.

Brialmontin II (**4**) was obtained as a white amorphous powder: ^1^H- and ^13^C-NMR data: (Table 2); (+)-ESI-MS *m*/*z* 395 [M + Na]^+^.

### 3.4. ^13^C-NMR and OR Calculations

The theoretical calculations of compound **1** were carried out using Gaussian 09.1. (Gaussian, Wallingford, CT, USA) Conformational analysis was initially performed using Discovery Studio 4.0 Client (Dassault systems, Velizy Villacoublay, France). The optimized conformation geometries, thermodynamic parameters, and populations of all conformations were provided in the Supporting Information. The conformers were optimized at the B3LYP/6-31G (d,p) level. Room temperature equilibrium populations were calculated according to Boltzmann distribution law. ^13^C-NMR shielding constants of compound **1** were calculated with the GIAO method at the MPW1PW91-SCRF/6-31G (d,p) level in acetone with PCM. The shielding constants obtained were converted into chemical shifts by referencing to TMS at 0 ppm (*δ*_calcd_ = σ_TMS_ − σ_calcd_), where the σTMS was the shielding constant of TMS calculated at the same level. The parameters a and b of the linear regression

$$\delta_{calc}=a\cdot +b;$$

the correlation coefficient, *R*^2^; the mean absolute error (MAE) defined as

∑n|δcalc−δexpt|n;

the corrected mean absolute error (CMAE), defined as

∑n|δcorr−δexpt|n,

where *δ_corr_ = (δ_calcd_ − b)/a* and therefore corrects for systematic errors were presented. Rotation calculations were carried at the B3LYP/6-31+G (d,p) and B3LYP/6-311++G (2d,p) level with PCM model in MeOH based on B3LYP/6-31G (d,p) optimized geometries optical to determine absolute stereochemistry of **1**.

### 3.5. Bioactive Assay of Gypmacrophin A

The acetylcholinesterase inhibitory effects were assayed by the enzyme-inhibitor approach using *S*-acetylthiocholine iodide as a substrate [21]. Compound **1** was dissolved in DMSO. The reaction mixture (in total, 200 μL) containing phosphate buffer (pH 8.0), test compound (50 μM), and acetyl cholinesterase (0.02 U/mL), was incubated for 20 min (37 °C). Then, the reaction was initiated by the addition of 40 μL of solution containing DTNB (0.625 mM) and acetylthiocholine iodide (0.625 mM) for AChE inhibitory activity assay, respectively. The hydrolysis of acetylthiocholine was monitored at 405 nm every 30 s for 1 h. Tacrine was used as positive control with final concentration of 0.333 μM. All of the reactions were performed in triplicate. The percentage inhibition was calculated as follows:

inhibition rate (%)=(E−S)E·100

where *E* is the activity of the enzyme without test compound and *S* is the activity of enzyme with test compound.

## 4. Conclusions

In summary, a rare sesterterpenoid and three depsides, rather than “triterpenoids”, were extracted from 1 g of materials from *G. macrophylla*, which provided new chemical evidence on research of its taxonomy and systematics. The structure and absolute configurations of gypmacrophin A were elucidated by spectroscopic analyses and computational methods. It showed weak inhibition of AchE with an IC_50_ value of 32.03 μM. The manuscripts can also explain the negative reaction of K (potassium hydroxide), C (calcium hypochlorite), P (*p*-phenylenediamine) with *G. macrophylla* recorded in the literature [15,16], since the depsides in *G. macrophylla* were polysubstituted by different levels of methyl or methoxy groups without any substitution of carboxylic group. From the perspective of the chemical angle, rarely do depsides have multisubstitution without carboxylic groups, partly indicating a special taxonomic status of *G. macrophylla* in lichens, which is now a single species in the genus of *Gypsoplaca* and family of *Gypsoplacaceae*.

## Figures and Tables

**Table 1:** Experimental and calculated spectroscopic data of gypmacrophin A (*δ* in ppm, recorded in acetone-*d*_6_).

Position	*δ*_H_-exp ^a^	*δ*_C_-exp ^b^	*δ*_C_-cal ^c^
1*β*	1.41 (overlapped, 1H)	40.9	40.1
1*α*	1.13 (d, *J* = 14.1 Hz, 1H)	--	--
2	1.99 (overlapped, 1H)	49.7	49.8
3	2.28 (m, 1H)	37.7	39.9
4*β*	1.29 (m, 1H)	32.4	33.2
4*α*	1.94 (m, 1H)	--	--
5*β*	2.12 (m, 1H)	38.8	40.4
5*α*	1.53 (dd, *J* = 13.0, 5.7 Hz, 1H)	--	--
6	--	83.3	84.7
7	--	24.2	27.4
8*β*	0.24 (dd, *J* = 8.5, 4.1 Hz, 1H)	16.4	16.7
8*α*	1.02 (overlapped, 1H)	--	--
9	0.54 (ddd, *J* = 11.4, 8.5, 5.5 Hz, 1H)	24.8	26.3
10	1.76 (t, *J* = 11.4 Hz, 1H)	38.8	40.2
11	--	39.7	42.6
12*β*	1.18 (dd, *J* = 12.7, 4.9 Hz, 1H)	39.4	38.9
12*α*	1.60 (overlapped, 1H)	--	--
13	4.91 (dd, *J* = 11.7, 4.9 Hz, 1H)	77.3	77.4
14	--	49.4	51.9
15	1.60 (m, 1H)	44.6	45.3
16	2.02 (overlapped, 1H)	42.2	42.9
17*β*	1.92 (overlapped, 1H)	28.7	30.1
17*α*	1.42 (m, 1H)	--	--
18	4.65 (t, *J* = 9.0 Hz, 1H)	81.1	81.9
19	0.83 (d, *J* = 7.4, 3H)	15.9	16.2
20	1.07 (s, 3H)	25.8	25.6
21	1.03 (s, 3H)	22.8	22.8
22	0.92 (s, 3H)	8.7	10.7
23	2.31 (m, 1H)	28.4	30.6
24	0.85 (d, *J* = 7.3, 3H)	23.2	23.2
25	0.86 (d, *J* = 7.3, 3H)	15.5	15.4
AcO-13	--	170.4	166.9
--	1.93 (s, 3H)	21.2	22.1
AcO-18	--	170.2	166.8
--	1.90 (s, 3H)	20.9	21.8
HO-6	2.96 (s, 1H)	--	--

^a^ recorded at ^1^H-NMR (600 Hz); ^b^ recorded at ^13^C-NMR (150 Hz); ^c^ calculation of ^13^C-NMR in acetone at the PW1PW91/6-31G (d,p) level.

**Table 2:** Spectroscopic data of brialmontins III (**2**), I (**3**) and II (**4**) (*δ* in ppm, recorded in acetone-*d*_6_).

Position	*δ*_H_ (2) ^a^	*δ*_C_ (2) ^b^	*δ*_H_ (3) ^a^	*δ*_C_ (3) ^b^	*δ*_H_ (4) ^a^	*δ*_C_ (4) ^b^
1	--	105.1	--	123.1	--	121.5
2	--	161.8	--	162.3	--	159.8
3	--	109.3	--	109.8	--	117.1
4	--	159.8	--	161.0	--	155.4
5	--	117.3	--	117.2	--	125.7
6	--	138.4	--	138.6	--	136. 0
7	--	171.5	--	171.0	--	167.1
8	2.66 (s, 3H)	19.5	2.64 (s, 3H)	19.3	2.14 (s, 3H)	17.3
9	2.23 (s, 3H)	12.4	2.23 (s, 3H)	12.6	2.17 (s, 3H)	12.5
10	2.16 (s, 3H)	8.7	2.16 (s, 3H)	9.3	2.31 (s, 3H)	9.8
1′	6.79 (s, 1H)	110.8	6.80 (s, 1H)	111.0	6.77 (s, 1H)	110.7
2′	--	156.8	--	156.6	--	156.8
3′	--	116.6	--	116.5	--	122.9
4′	--	149.2	--	149.2	--	149.6
5′	--	136.1	--	136.2	--	127.1
6′	--	121.0	--	121.0	--	133.9
7′	2.31 (s, 3H)	20.2	2.31 (s, 3H)	20.2	2.23 (s, 3H)	20.3
8′	2.02 (s, 3H)	12.6	2.02 (s, 3H)	12.7	2.27 (s, 3H)	12.7
9′	1.99 (s, 3H)	9.7	1.99 (s, 3H)	9.8	2.34 (s, 3H)	9.9
CH_3_O-2′	3.85 (s, 3H)	56.0	3.80 (s, 3H)	56.1	3.74 (s, 3H)	56.0
R_2_O-2	11.56 (s, 1H)		11.02 (s, 1H)		3.84 (s, 3H)	62.1
R_1_O-4	8.18 (s, 1H)		3.74 (s, 3H)	60.3	3.81 (s, 3H)	60.3

^a^ recorded at ^1^H-NMR (800 Hz); ^b^ recorded at ^13^C-NMR (200 Hz).

## Supplementary Materials

The following ^1^H-NMR, ^13^C-NMR, 2D-NMR, HR-ESI-MS, OR, CD, UV and IR spectra are available as supporting data. Supplementary materials are available online.

## References

[1] Tian W., Deng Z., Hong K. (2017). The biological activities of sesterterpenoid-Type ophiobolins. Mar. Drugs.

[2] Evidente A., Kornienko A., Lefranc F., Cimmino A., Dasari R., Evidente M., Mathieu V., Kiss R. (2015). Sesterterpenoids with anticancer activity. Curr. Med. Chem..

[3] Baquero F., Coque T.M., de la Cruz F. (2011). Ecology and evolution as targets: The need for novel eco-evo drugs and strategies to fight antibiotic resistance. Antimicrob. Agents Chemother..

[4] García A., Bocanegra-García V., Palma-Nicolás J.P., Rivera G. (2012). Recent advances in antitubercular natural products. Eur. J. Med. Chem..

[5] Stowe S.D., Richards J.J., Tucker A.T., Thompson R., Melander C., Cavanagh J. (2011). Anti-Biofilm compounds derived from marine sponges. Mar. Drugs.

[6] Wang L., Yang B., Lin X.P., Zhou X.F., Liu Y. (2013). Sesterterpenoids. Nat. Prod. Rep..

[7] Liu Z., Chen Y., Chen S., Liu Y., Lu Y., Chen D., Lin Y., Huang X., She Z. (2016). Aspterpenacids A and B, two sesterterpenoids from a mangrove endophytic fungus *Aspergillus terreus* H010. Org. Lett..

[8] Hanson J.R. (1992). The sesterterpenoids. Nat. Prod. Rep..

[9] Okada M., Matsuda Y., Mitsuhashi T., Hoshino S., Mori T., Nakagawa K., Quan Z., Qin B., Zhang H., Hayashi F. (2016). Genome-based discovery of an unprecedented cyclization mode in fungal sesterterpenoid biosynthesis. J. Am. Chem. Soc..

[10] Matsuda Y., Mitsuhashi T., Lee S., Hoshino M., Mori T., Okada M., Zhang H.P., Hayashi F., Fujita M., Abe I. (2016). Astellifadiene: Structure determination by NMR spectroscopy and crystalline sponge method, and elucidation of its biosynthesis. Angew. Chem. Int. Ed..

[11] Lutzoni F., Pagel M., Reeb V. (2001). Major fungal lineages are derived from lichen symbiotic ancestors. Nature.

[12] Mittermeier K.V., Schmitt N., Volk P.L., Suárez P.J., Beck A., Eisenreich W. (2015). Metabolic profiling of alpine and ecuadorian lichens. Molecules.

[13] Zhang Y.Y., Wang X.Y., Liu D., Shi H.X., Ye X., Yang M.X., Wang L.S. (2016). The genus *Bulbothrix* (*Parmeliaceae*) in China. Lichenologist.

[14] Wang X.Y., Goffinet B., Liu D., Liang M.M., Shi H.X., Zhang Y.Y., Zhang J., Wang L.S. (2015). Taxonomic study of the genus *Anzia* (*Lecanorales*, lichenized Ascomycota) from Hengduan Mountains, China. Lichenologist.

[15] Timdal E. (1990). Gypsoplacaceae and *Gypsoplaca*, a new family and genus of squamiform lichens. Bibl. Lichenol..

[16] Abdulla A., Hurnisa X., Reyim M., Adilijiang A. (2015). A new record of lichen family from Xinjiang. Arid Zone Res..

[17] Zhao Z.-Z., Chen H.-P., Wu B., Zhang L., Li Z.-H., Feng T., Liu J.-K. (2017). Matsutakone and Matsutoic Acid, Two (Nor)steroids with unusual skeletons from the edible mushroom *Tricholoma matsutake*. J. Org. Chem..

[18] Elix J.A., Barclay C.E., David F., Griffin F.K., Hill A.M., Mcconnell D.B., Wardlaw J.H. (1993). Synthesis of further lichen depsides. Aust. J. Chem..

[19] Seo C., HanYim J., Kum Lee H., Oh H. (2011). PTP1B inhibitory secondary metabolites from the Antarctic lichen *Lecidella carpathica*. Mycology.

[20] Huang X., Huang H., Li H., Sun X., Huang H., Lu Y., Lin Y., Long Y., She Z. (2013). Asperterpenoid A, a new sesterterpenoid as an inhibitor of *Mycobacterium tuberculosis* protein tyrosine phosphatase B from the culture of *Aspergillus* sp. 16–5c. Org. Lett..

[21] Ellman G.L., Courtney K.D., Andres V., Featherstone R.M. (1961). A new and rapid colorimetric determination of acetylcholinesterase activity. Biochem. Pharmacol..

