# Supplementary material for: Gypmacrophin A, a Rare Pentacyclic Sesterterpenoid, Together with Three Depsides, Functioned as New Chemical Evidence for Gypsoplaca macrophylla (Zahlbr.) Timdal Identification
Source: Molecules. 2017 Oct 9;22(10):1675. doi: 10.3390/molecules22101675 (PMC6151673; doi:10.3390/molecules22101675)
Supplement: Supplementary file 1 [file molecules-22-01675-s001.pdf]

Supporting Information

Gypmacrophin A, a Rare Pentacyclic Sesterterpenoid, Together with Three Depsides, Functioned as New Chemical Evidence for *Gypsoplaca macrophylla* (Zahlbr.) Timdal Identification

Yuan-Fei Zhou[1,2], Hai-Xia Shi[3], Kun Hu[1,2], Jian-Wei Tang[1,2], Xing-Ren Li[1,2], Xue Du[1], Han-Dong Sun[1], Li-Song Wang[3,*], and Jian-Xin Pu[1,*]

[1] State Key Laboratory of Phytochemistry and Plant Resources in West China, Kunming Institute of Botany, Chinese Academy of Sciences, Kunming 650201, China; zhouyuanfei@mail.kib.ac.cn (Y.-F.Z.); hukun@mail.kib.ac.cn (K.H.); tangjianwei@mail.kib.ac.cn (J.-W.T.); lixingren@mail.kib.ac.cn (X.-R.L.); duxue@mail.kib.ac.cn (X.D.); hdsun@mail.kib.ac.cn (H.-D.S.); pujianxin@mail.kib.ac.cn (J.-X.P.)

[2] Kunming College of Life Sciences, University of Chinese Academy of Sciences, Beijing 100039, China

[3] Key Laboratory for Plant Biodiversity and Biogeography of East Asia, Kunming Institute of Botany, Chinese Academy of Sciences, Kunming 650201, China; shihaixia@mail.kib.ac.cn (S.-H.X.); wanglisong@mail.kib.ac.cn (L.-S.W.)

* Correspondence: pujianxin@mail.kib.ac.cn (J.-X.P.); wanglisong@mail.kib.ac.cn (L.-S.W.)
Tel.: +86-871-65223616 (J.-X.P.)

Contents of Supporting Information

NO.	Contents	Pages
1	**Figure S1.** Habit of ***Gypsoplaca macrophylla*** **(Zahlbr.) Timdal**	1
2	**Figure S2.** ^{1}H NMR spectrum of Gypmacrophin A in acetone-d_6	2
3	**Figure S3.**Expand ^{1}H NMR spectrum of Gypmacrophin A in acetone-d_6	3
4	**Figure S4.** ^{13}C NMR spectrum of Gypmacrophin A in acetone-d_6	4
5	**Figure S5.** Expand ^{13}C NMR spectrum of Gypmacrophin A in acetone-d_6	5
6	**Figure S6.** HSQC spectrum of Gypmacrophin A in acetone-d_6	6
7	**Figure S7.**Expand HSQC spectrum of Gypmacrophin A in acetone-d_6	7
8	**Figure S8.**HMBC spectrum of Gypmacrophin A in acetone-d_6	8
9	**Figure S9.**Expand HMBC spectrum of Gypmacrophin A in acetone-d_6	9
10	**Figure S10.**^{1}H-^{1}H COSY spectrum of Gypmacrophin A in acetone-d_6	10
11	**Figure S11**. Expand ^{1}H-^{1}H COSY spectrum of Gypmacrophin A in acetone-d_6	11
12	**Figure S12.**HSQC-TOCSY spectrum of Gypmacrophin A in acetone-d_6	12
13	**Figure S13**.Expand HSQC-TOCSY spectrum of Gypmacrophin A in acetone-d_6	13
14	**Figure S14.**ROESY spectrum of Gypmacrophin A in acetone-d_6	14
15	**Figure S15.**Expand ROESY spectrum of Gypmacrophin A in acetone-d_6	15
16	**Figure S16.**HRESIMS spectrum of Gypmacrophin A	16
17	**Figure S17.** UV spectrum of Gypmacrophin A	17
18	**Figure S18.** OR report of Gypmacrophin A	17
19	**Figure S19.** CD report of Gypmacrophin A	18
20	**Figure S20.** IR spectrum of Gypmacrophin A	18
21	**Figure S21.** ^{1}H NMR spectrum of Brialmontin Ⅲ in acetone-d_6	19
22	**Figure S22.** ^{13}C NMR spectrum of Brialmontin Ⅲ in acetone-d_6	20
23	**Figure S23.** HSQC spectrum of Brialmontin Ⅲin acetone-d_6	21
24	**Figure S24**. Expand HSQC spectrum of Brialmontin Ⅲin acetone-d_6	22
25	**Figure S25.** HMBC spectrum of Brialmontin Ⅲin acetone-d_6	23
26	**Figure S26.** Expand HMBC spectrum of Brialmontin Ⅲin acetone-d_6	24

**27**	**Figure S27.** ROESY spectrum of Brialmontin Ⅲin acetone-*d6*	**25**
**28**	**Figure S28.**HRESIMS spectrum of Brialmontin Ⅲ	**26**
**29**	**Figure S29.** IR spectrum of Brialmontin Ⅲ	**27**
**30**	**Figure S30.** UV spectrum of Gypmacrophin A	**27**
**31**	**Figure S31.** ^{1}H NMR spectrum of Brialmontin Ⅰ in acetone-*d6*	**28**
**32**	**Figure S32.** ^{13}C NMR spectrum of Brialmontin Ⅰ in acetone-*d6*	**29**
**33**	**Figure S33.** Positive ESI spectrum of Brialmontin Ⅰ	**30**
**34**	**Figure S34.** ^{1}H NMR spectrum of BrialmontinⅡin acetone-*d6*	**31**
**35**	**Figure S35.** ^{13}C NMR spectrum of BrialmontinⅡin acetone-*d6*	**32**
**36**	**Figure S36.** Positive ESI spectrum of BrialmontinⅡ	**33**
**37**	Bioactive assay of Gypmacrophin A	**34**
**38**	^{13}C NMR and OR calculations	**35**
**39**	Optimized conformers of 1(**Figure S37, Table S1**)	**36**
**40**	Results of 13C NMR calculations of 1(**Figure S38, Table S2**)	**37**
**41**	Results of OR calculations of 1(**Table S3**)	**39**
**42**	Z-matrix of 1 (1A and 1B) in gas phase at B3LYP/6-31G(d) level (**Table S4**)	**40**

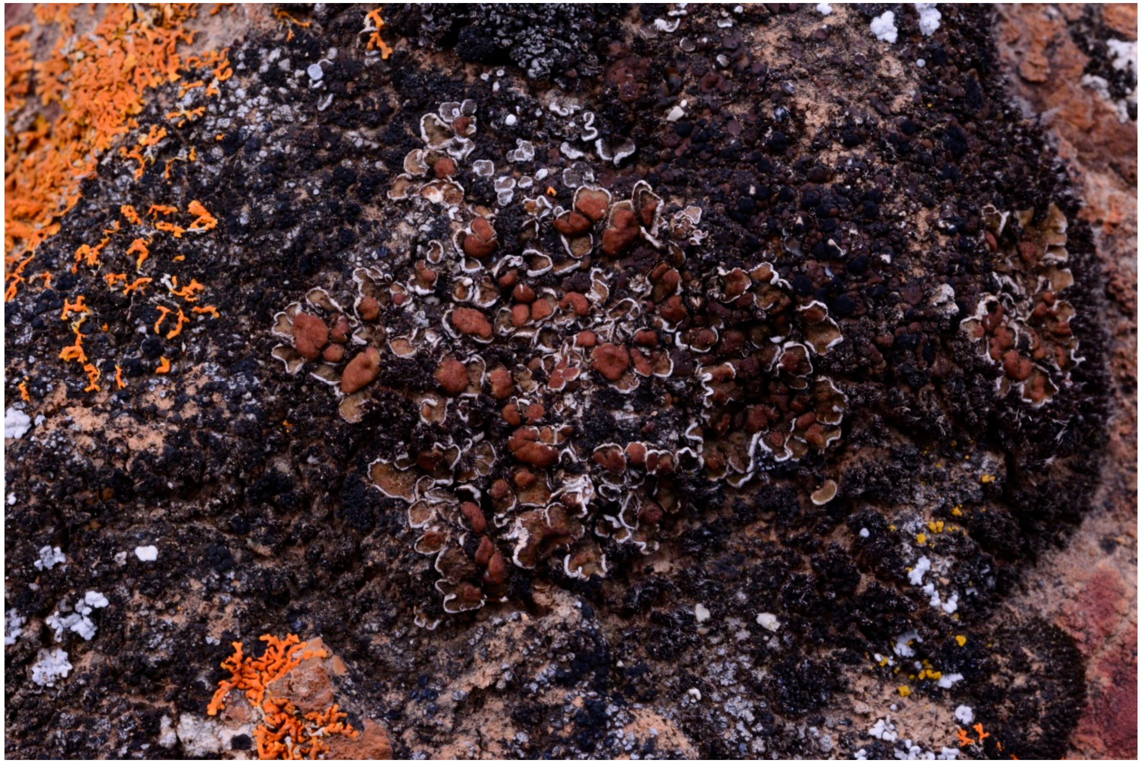

Figure S1. Habit of *Gypsoplaca macrophylla* (Yunnan Prov., Deqin Co., Baimaxueshan Mt., 4400 alt., on soil, Wang Li-song et al. 15-49586, in KUN-L-52617).

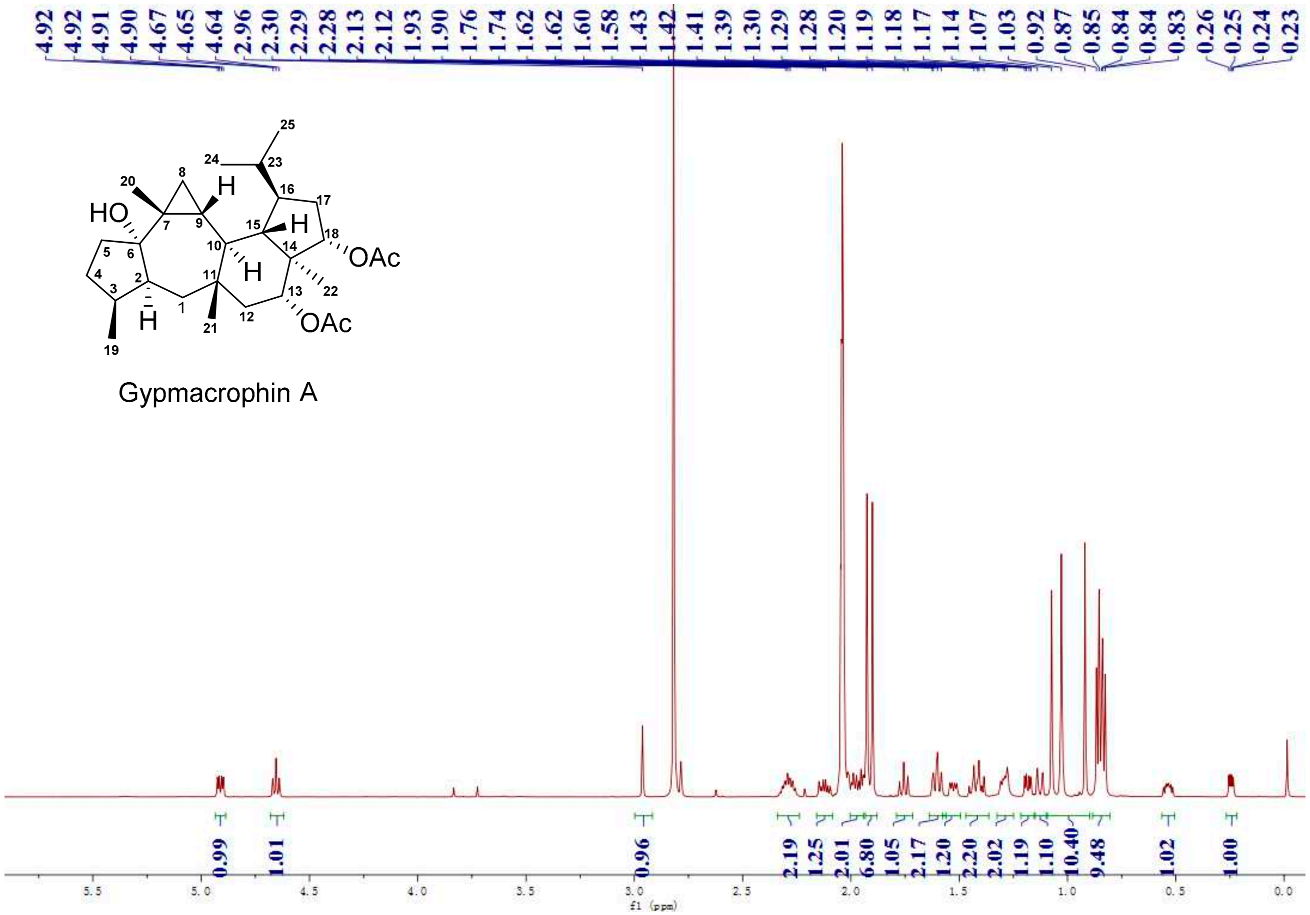

Figure S2. ^{1}H NMR spectrum of Gypmacrophin A in acetone-d_6.

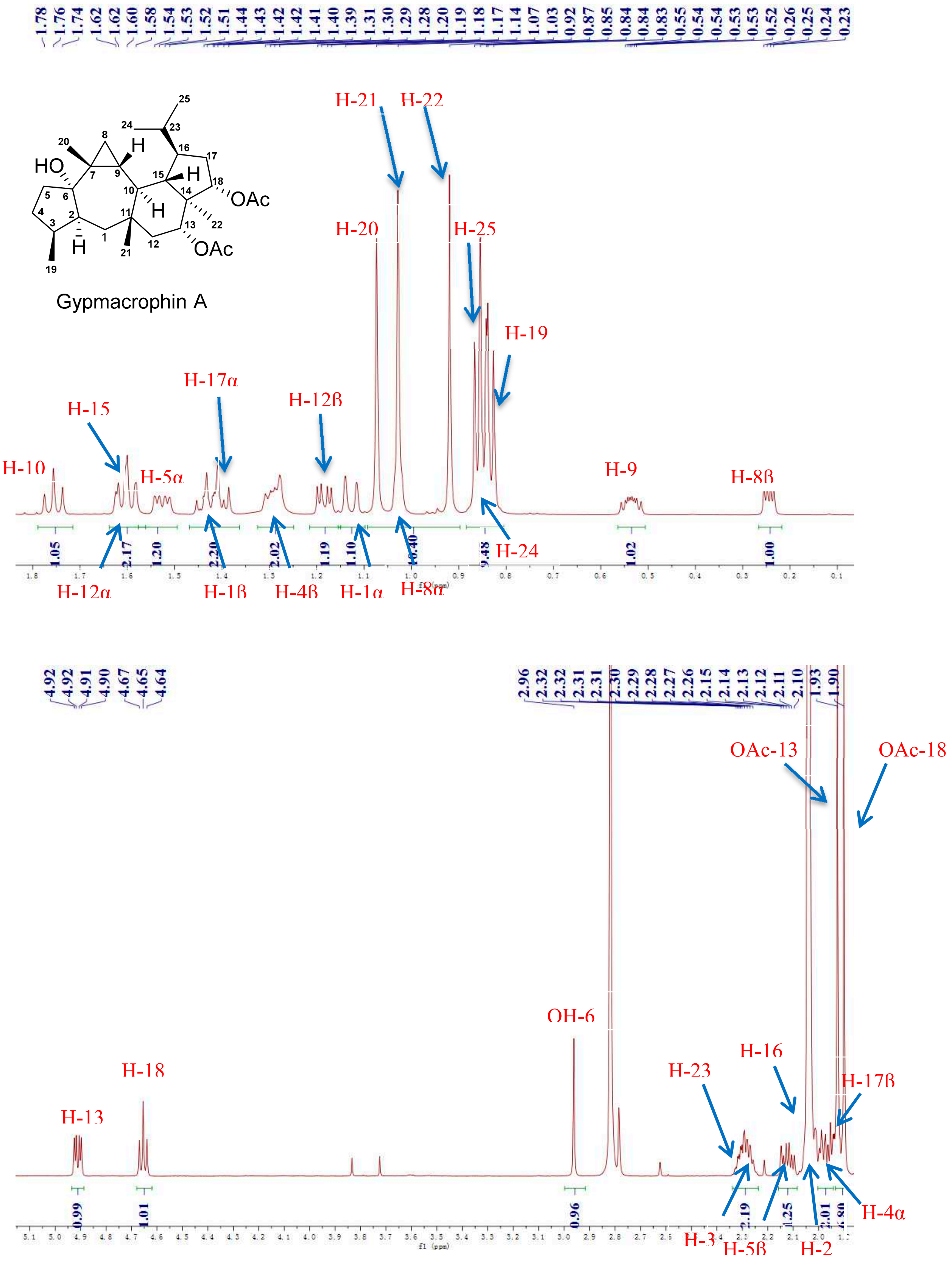

Figure S3.Expand ^{1}H NMR spectrum of Gypmacrophin A in acetone-*d*6.

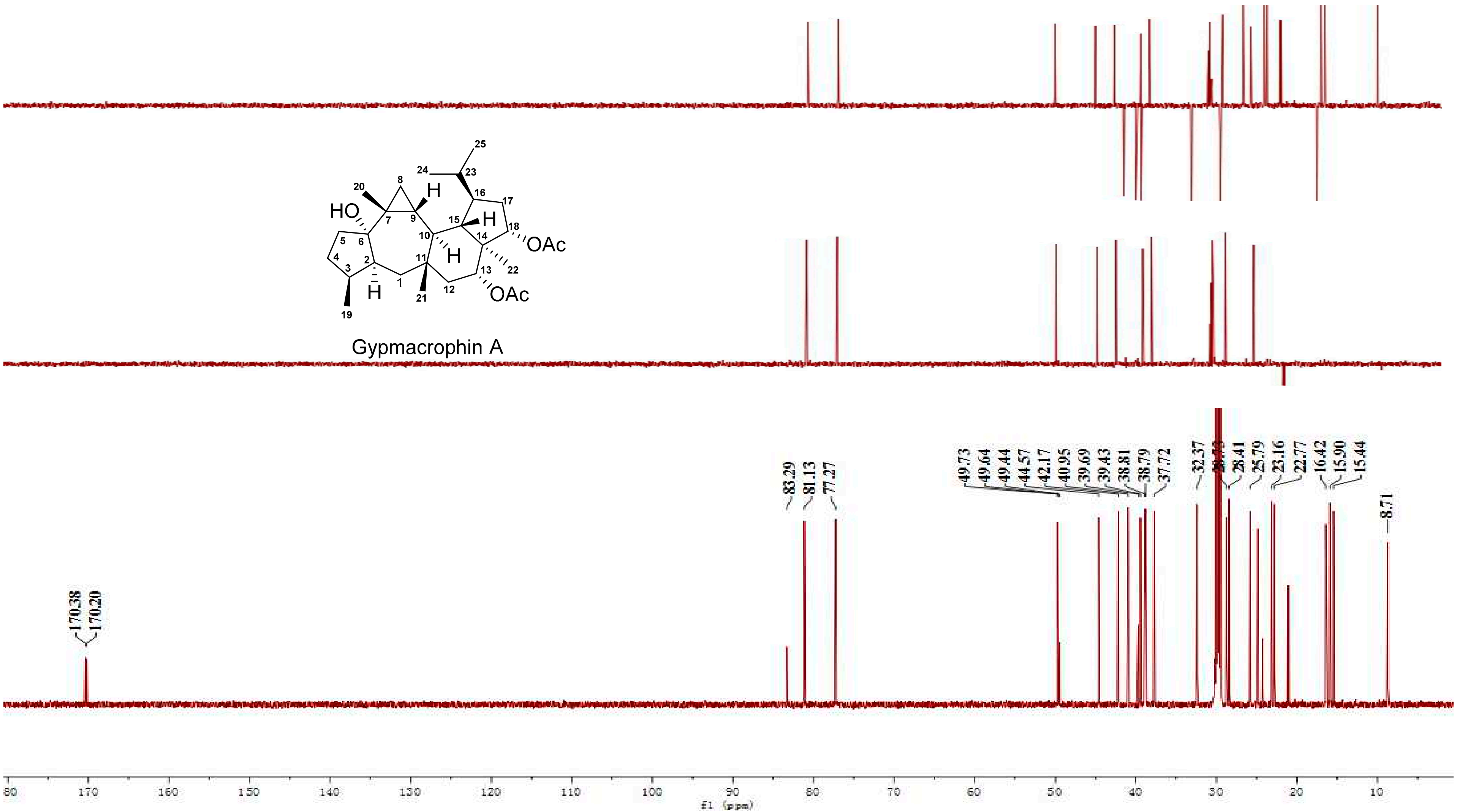

Figure S4. ^{13}C NMR spectrum of Gypmacronoid A in acetone-d_6.

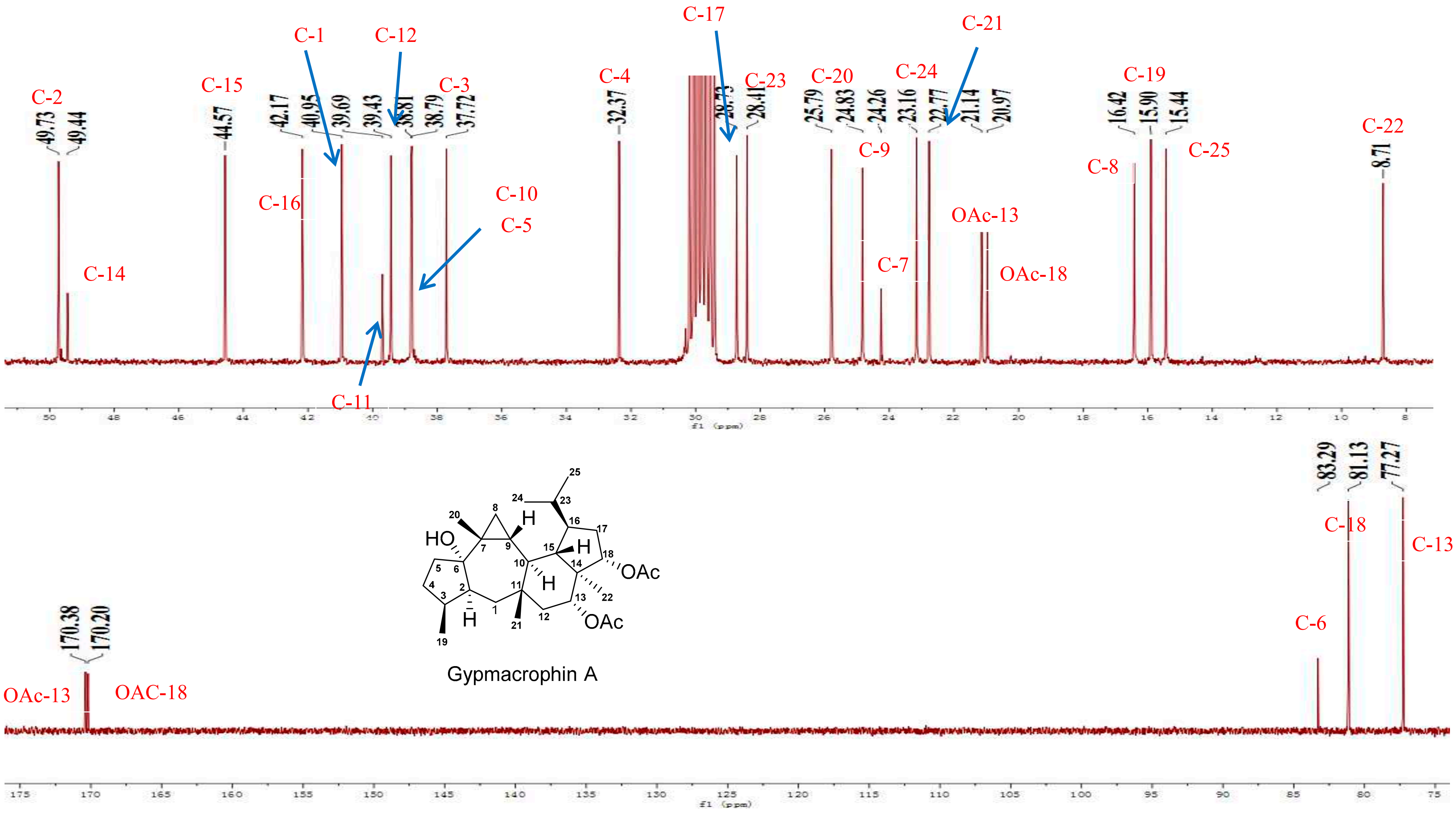

Figure S5. Expand ^{13}C NMR spectrum of Gypmacronoid A in acetone-d_6.

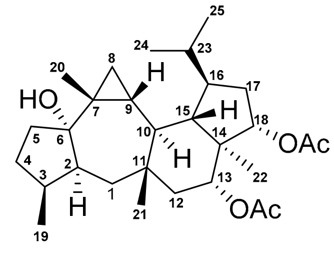

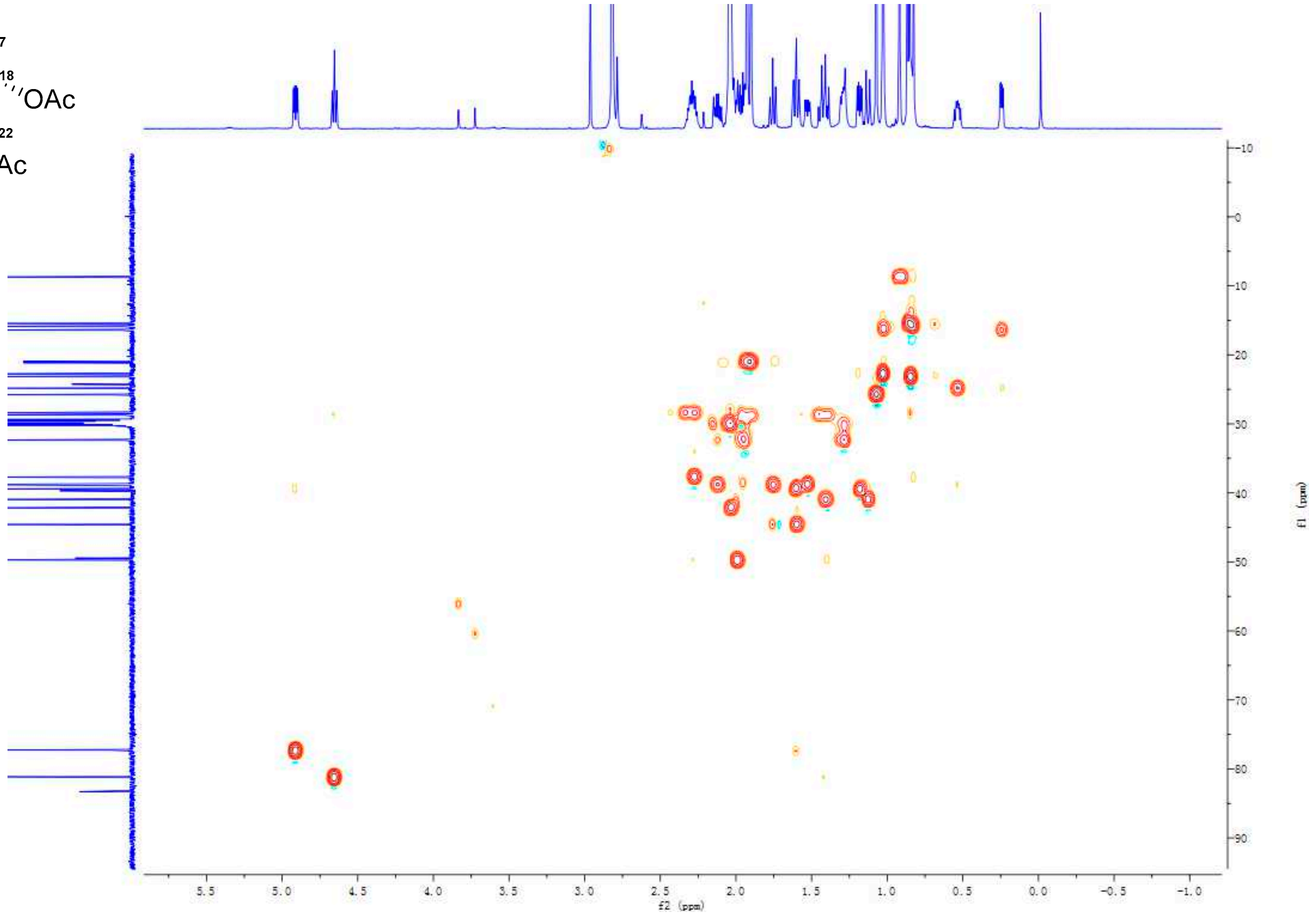

Figure S6. HSQC spectrum of Gypmacrophin A in acetone-d_6.

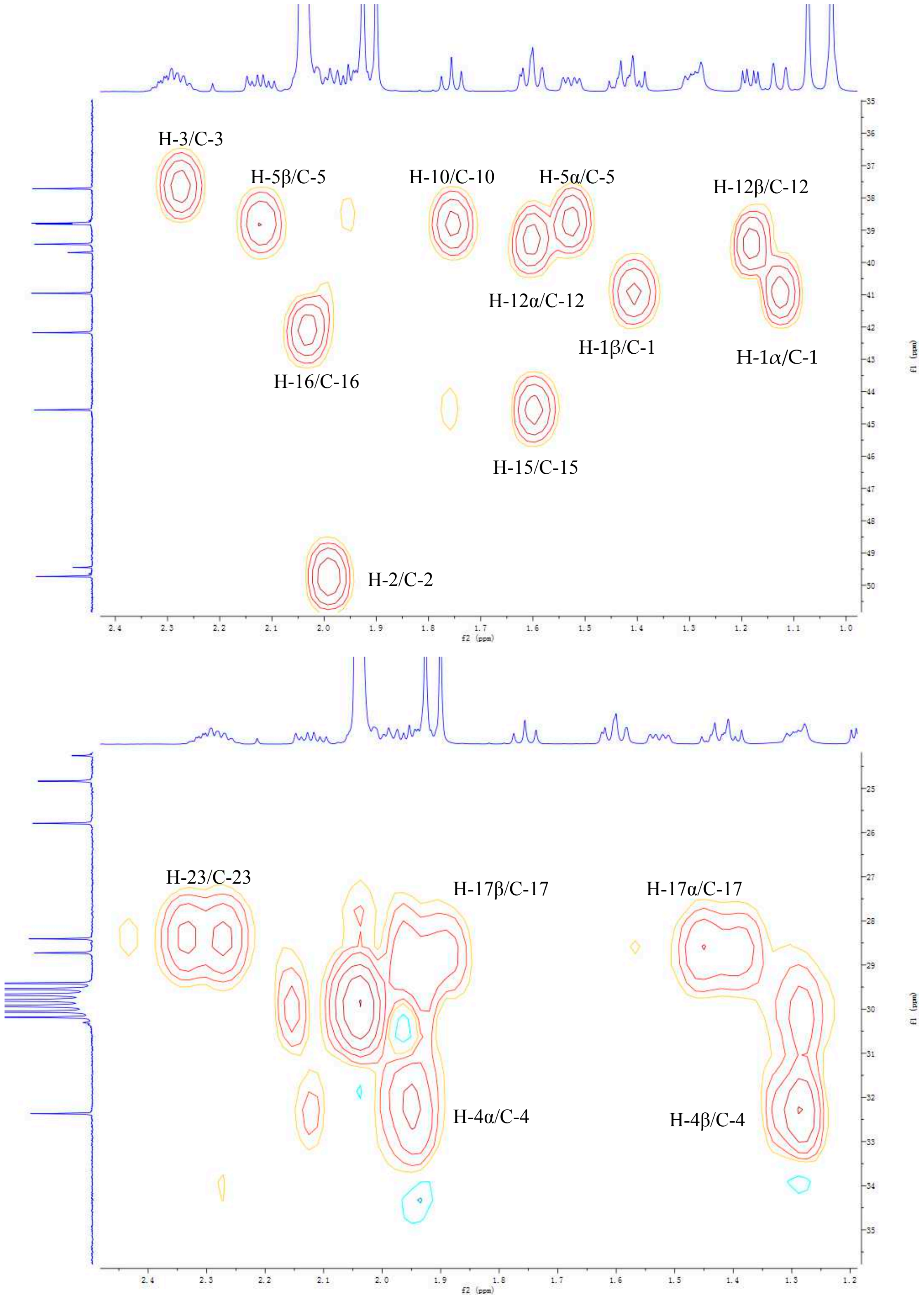

Figure S7. Expand HSQC spectrum of Gypmacrophin A in acetone-*d*6.

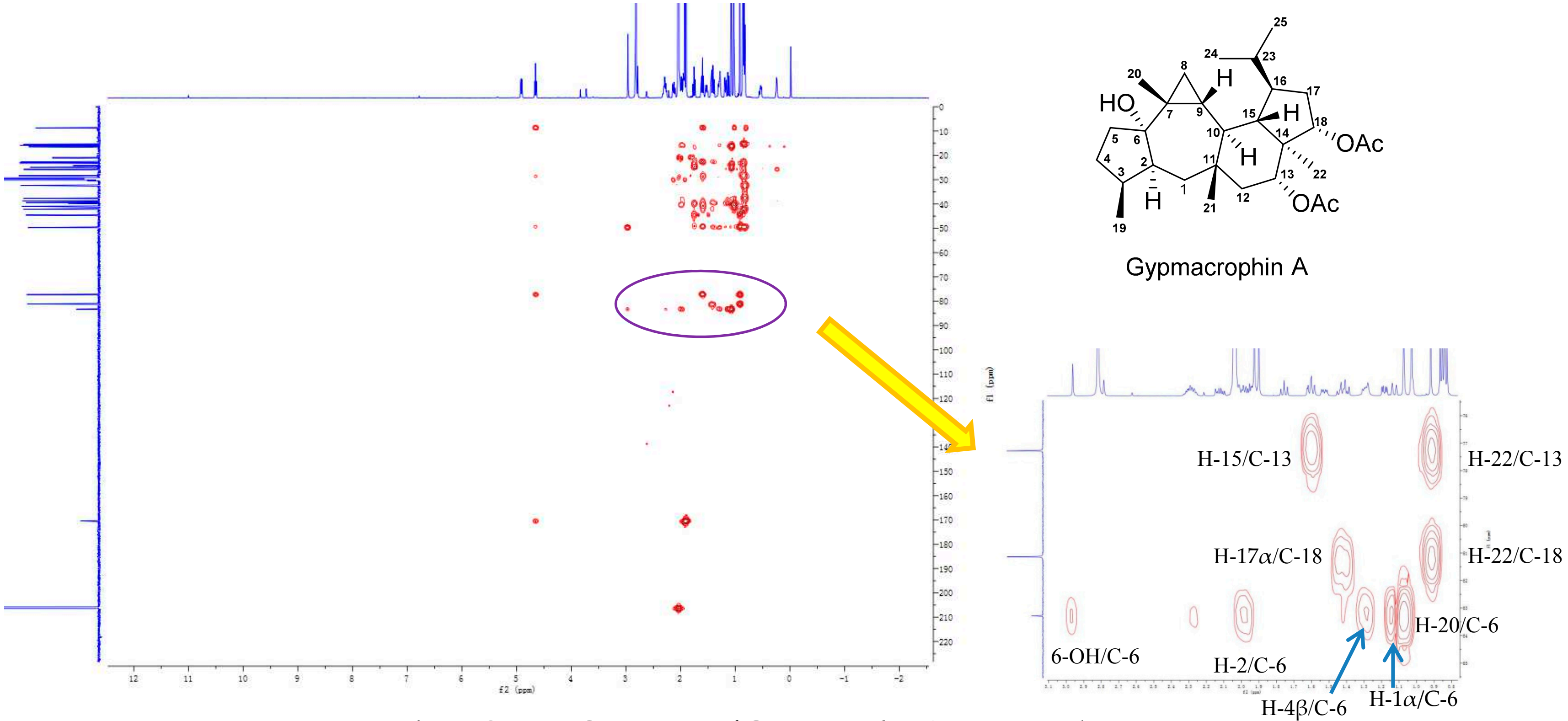

Figure S8. HMBC spectrum of Gypmacrophin A in acetone-d_6.

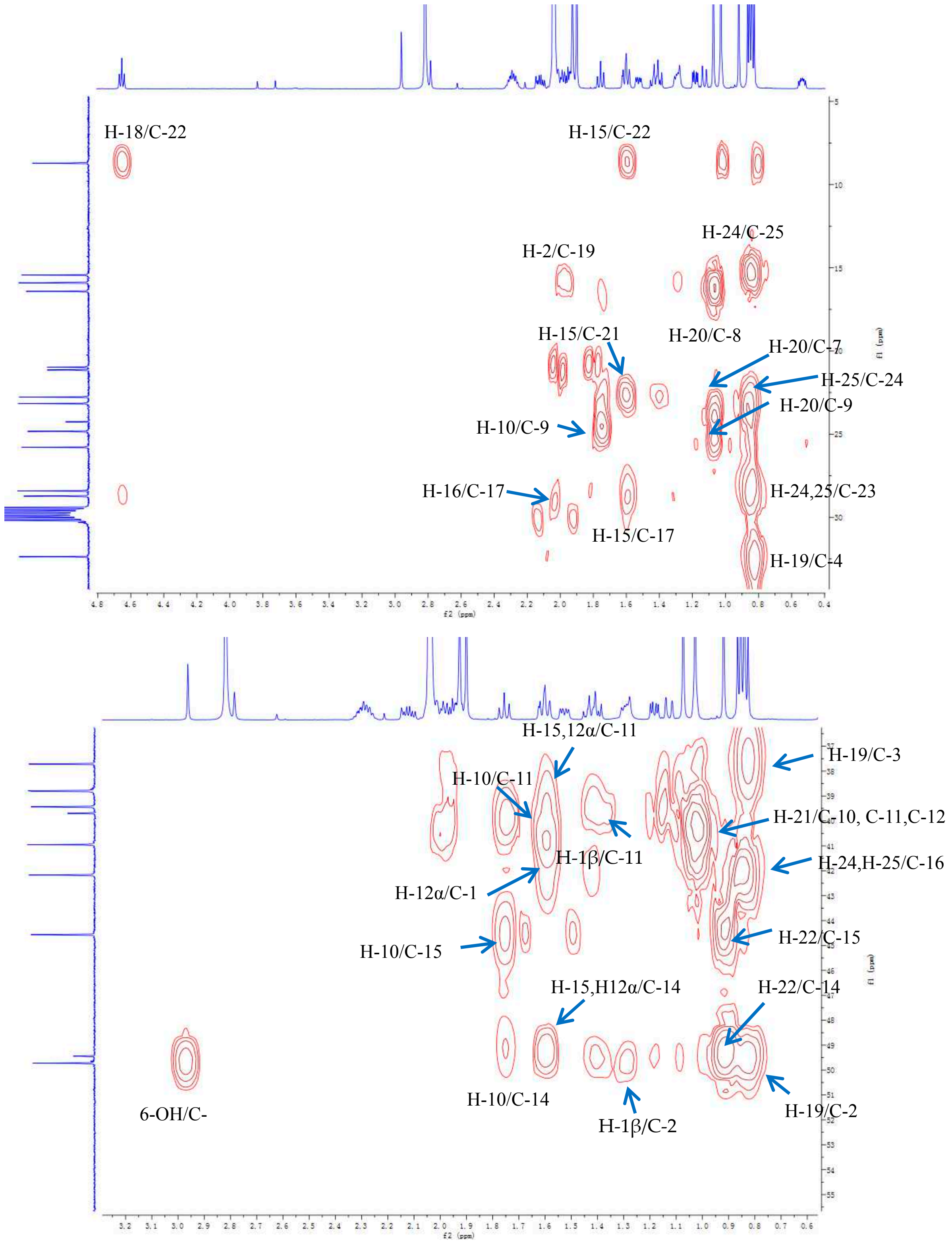

Figure S9. Expand HMBC spectrum of Gypmacrophin A in acetone-*d*6.

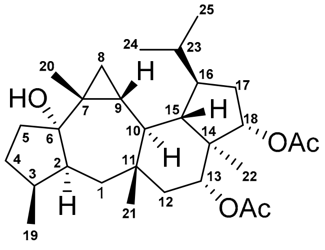

Gypmacrophin A

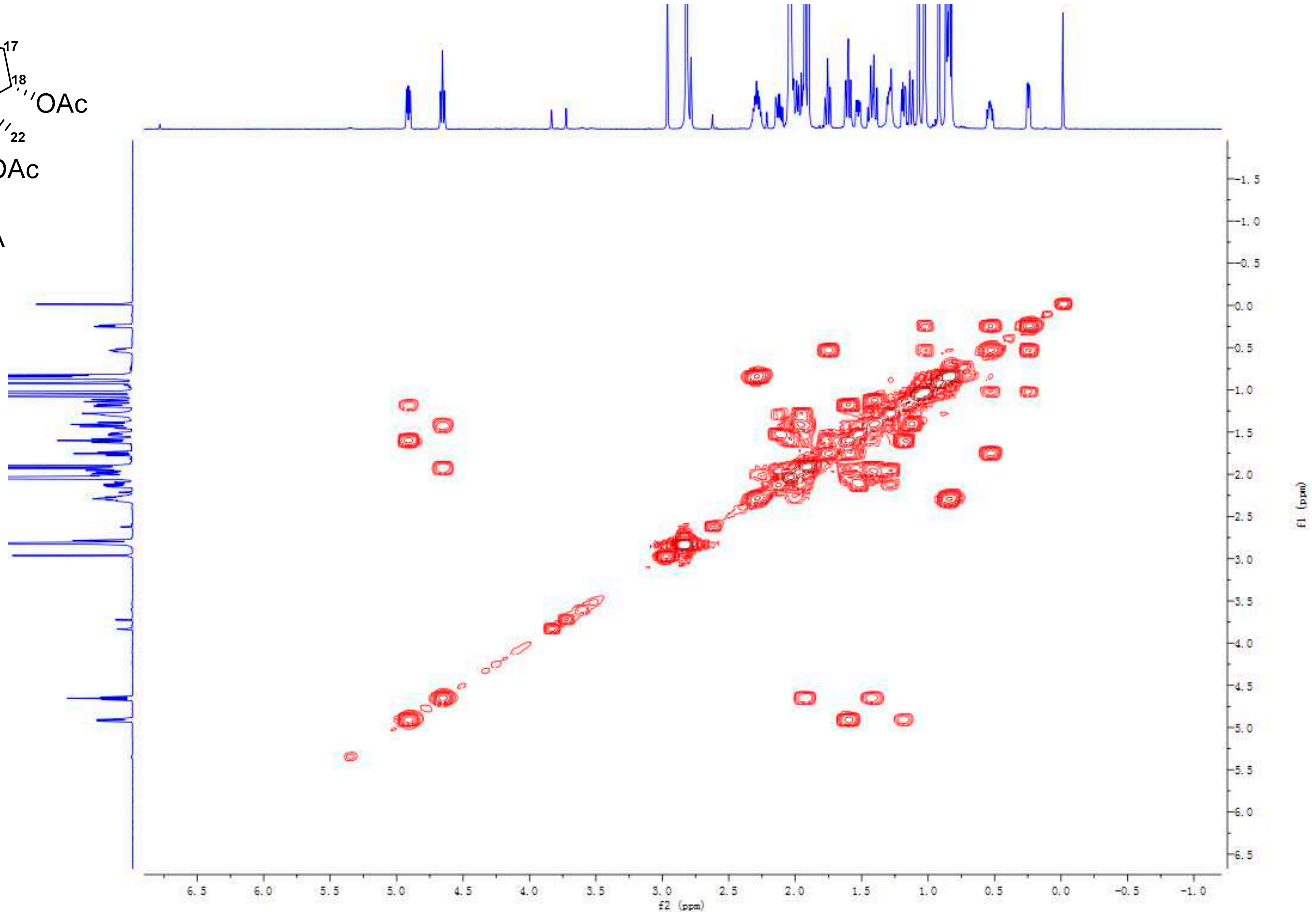

Figure S10.^{1}H-^{1}H COSY spectrum of Gypmacrophin A in acetone-*d6*.

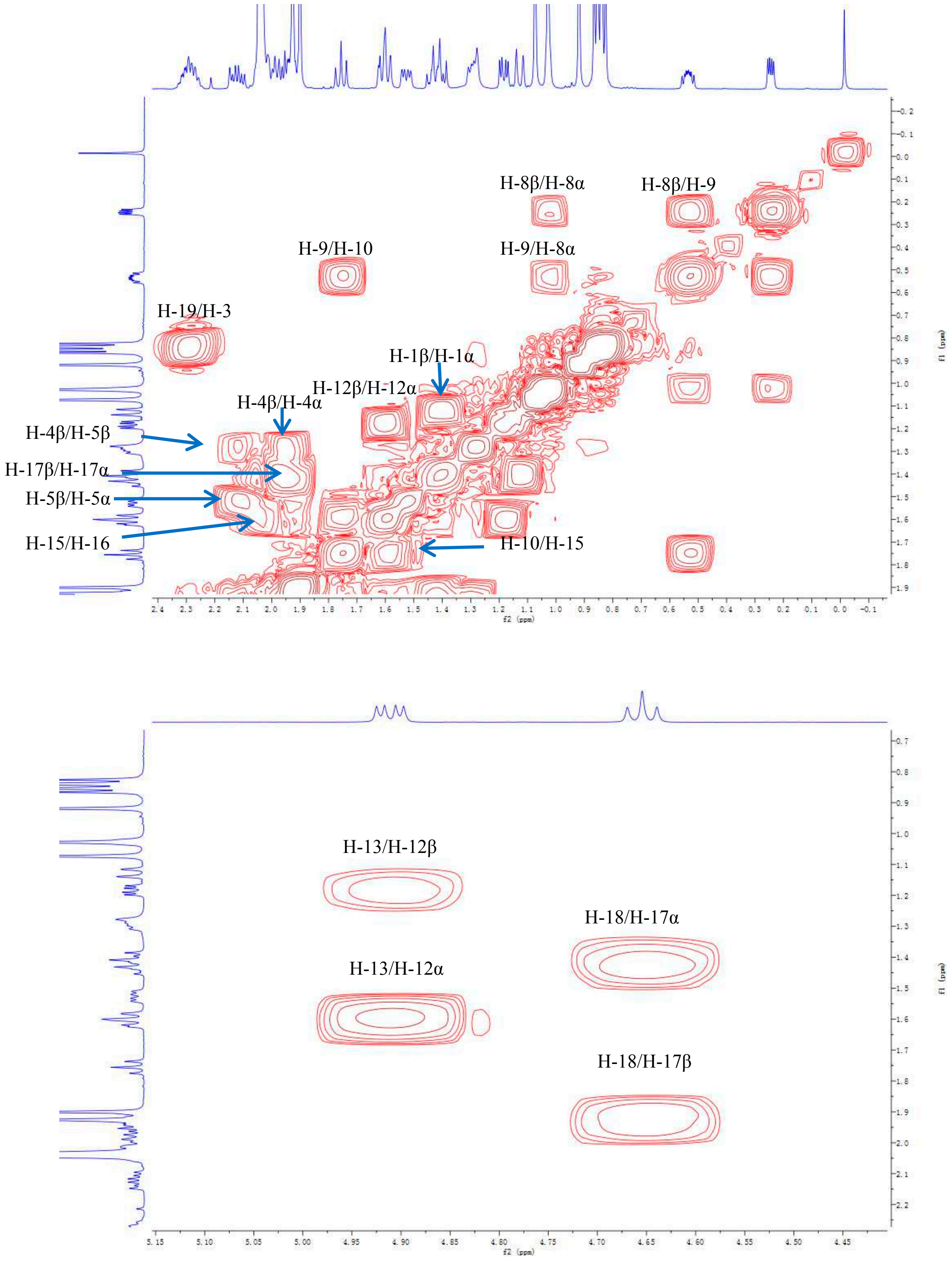

Figure S11.Expand ^{1}H-^{1}H COSY spectrum of Gypmacrophin A in acetone-d_6.

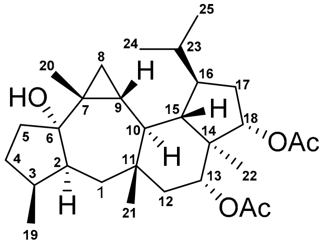

Gypmacrophin A

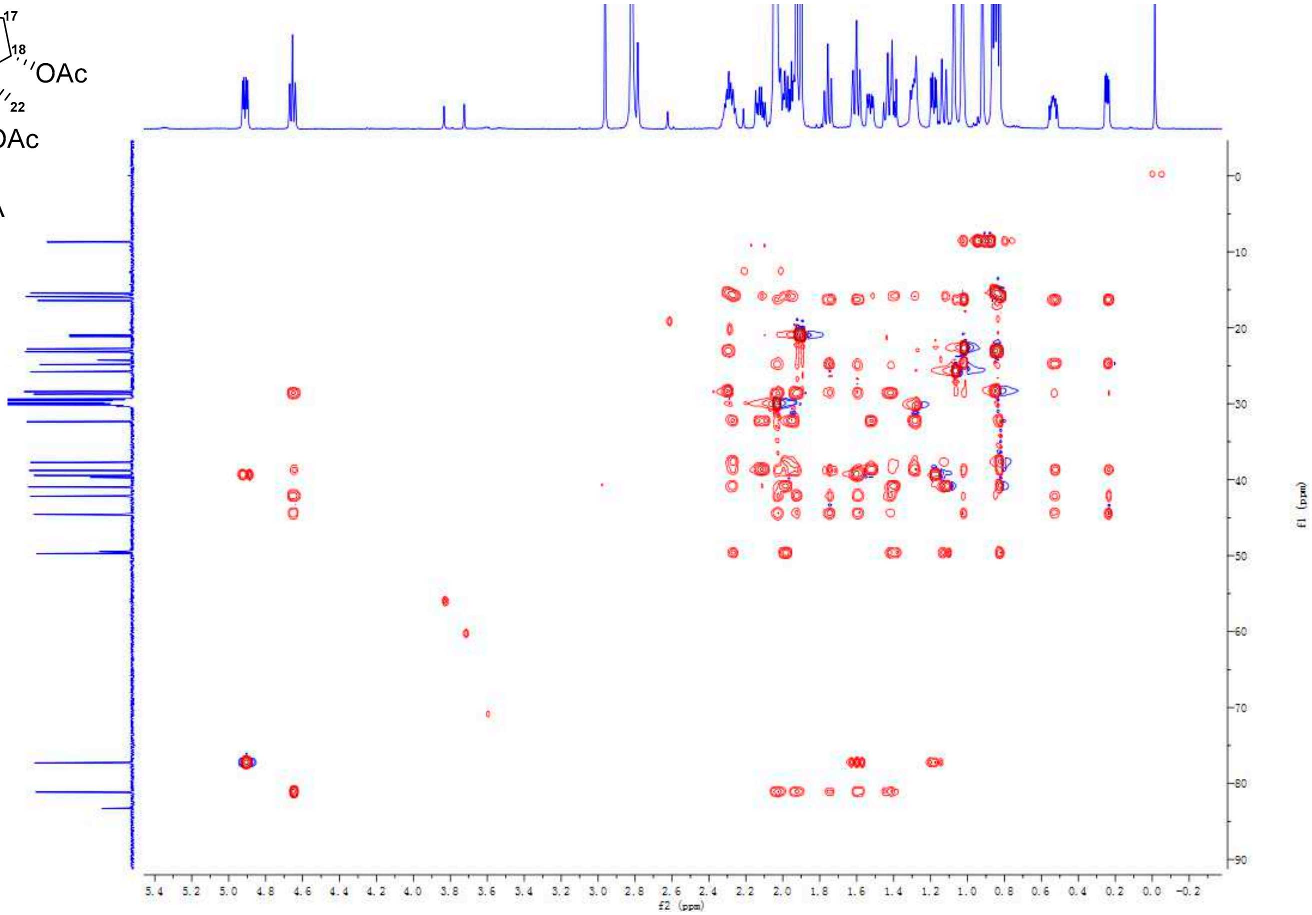

Figure S12.HSQC-TOCSY spectrum of Gypmacrophin A in acetone-d_6.

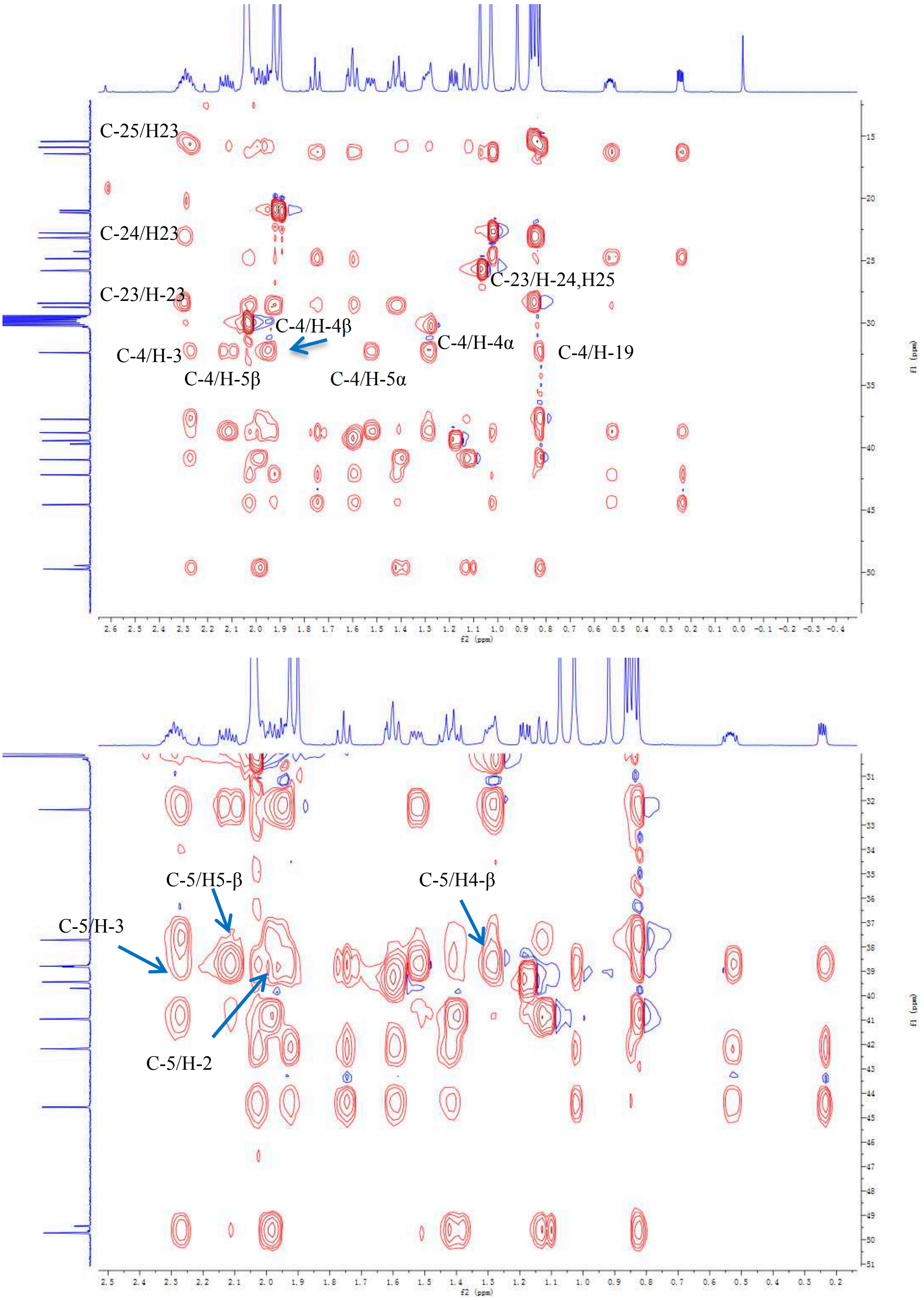

Figure S13.Expand HSQC-TOCSY spectrum of Gypmacrophin A in acetone-d_6.

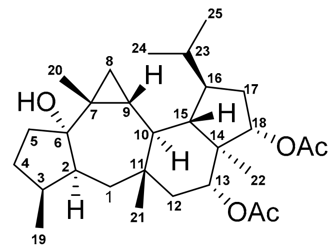

Gypmacrophin A

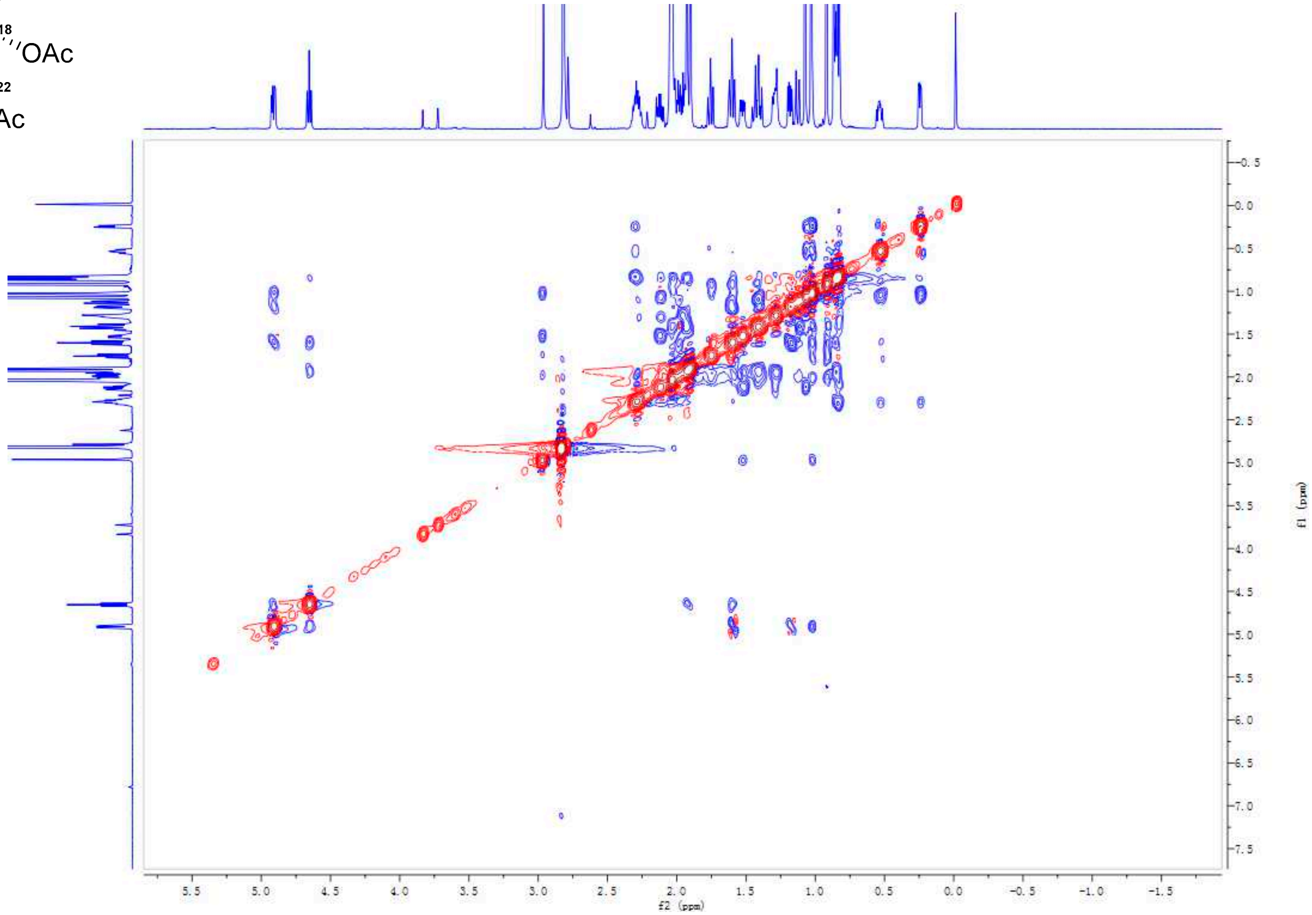

Figure S14.ROESY spectrum of Gypmacrophin A in acetone-*d*6.

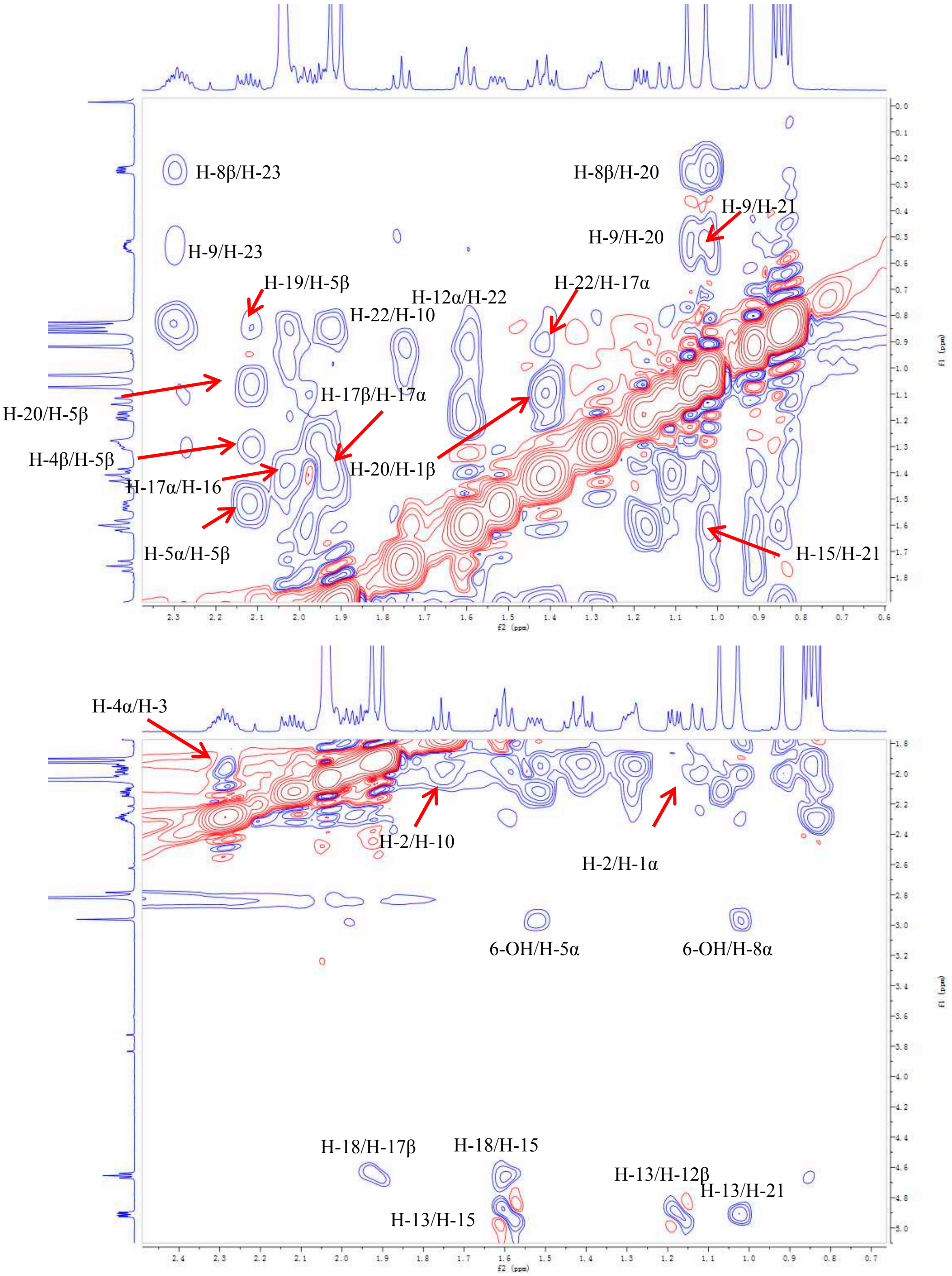

Figure S15. Expand ROESY spectrum of Gypmacrophin A in acetone-*d*6.

Qualitative Analysis Report

**Data Filename**	SDY-djx-2.d	**Sample Name**	SDY-djx-2
**Sample Type**	Sample	**Position**	P1-C6
**Instrument Name**	Instrument 1	**User Name**	
**Acq Method**	SIBU.m	**Acquired Time**	9/6/2016 11:03:07 AM
**IRM Calibration Status**	[redacted]	**DA Method**	ESI+.m
**Comment**			

Sample Group **Info.**
Acquisition SW 6200 series TOF/6500 series
Version Q-TOF B.05.01 (B5125.2)

User Spectra

Fragmentor Voltage	Collision Energy	Ionization Mode
135	0	ESI

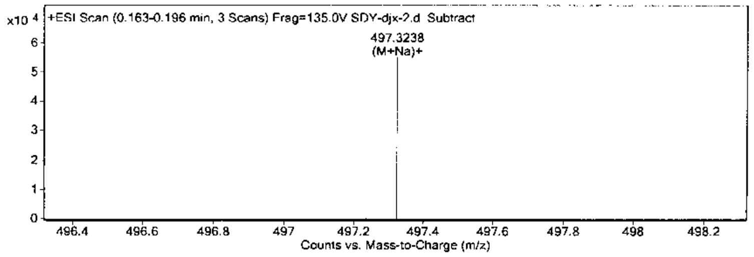

Peak List

m/z	z	Abund	Formula	Ion
492.3684	1	40334.05		
493.3715	1	13024.71		
497.3238	1	55149.73	C29 H46 O5	(M+Na)+
498.3271	1	17019.93	C29 H46 O5	(M+Na)+
513.2975	1	25618.65		
514.3008	1	8237.81		
647.4137	1	7775.78		
971.6583	1	8941.44		

Formula Calculator Element Limits

Element	Min	Max
C	3	60
H	0	120
O	0	30

Formula Calculator Results

Formula	CalculatedMass	CalculatedMz	Mz	Diff. (mDa)	Diff. (ppm)	DBE
C29 H46 O5	474.3345	497.3237	497.3238	0.0	-0.1	7.0000

--- End Of Report ---

Agilent Technologies Page 1 of 1 Printed at: 11:04 AM on: 9/6/2016

Figure S16.HRESIMS spectrum of Gypmacrophin A.

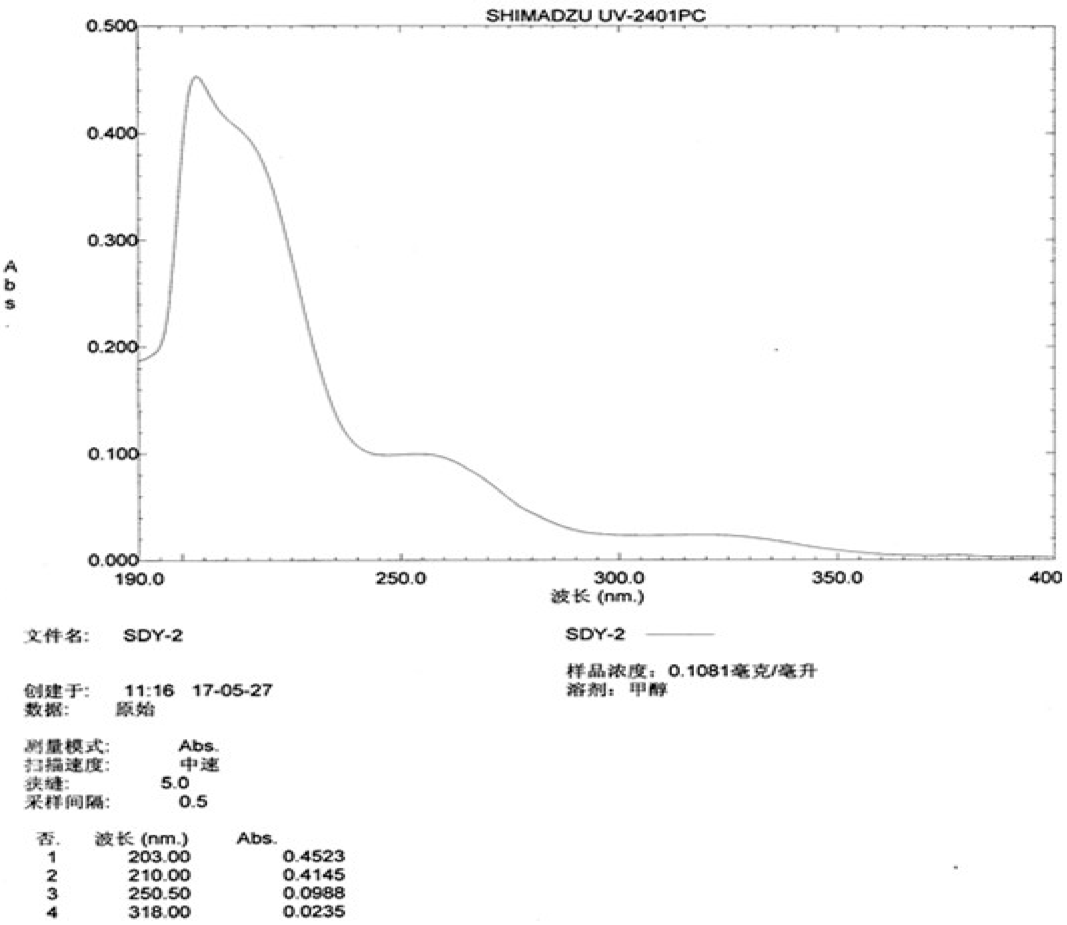

Figure S17. UV spectrum of Gypmacrophin A.

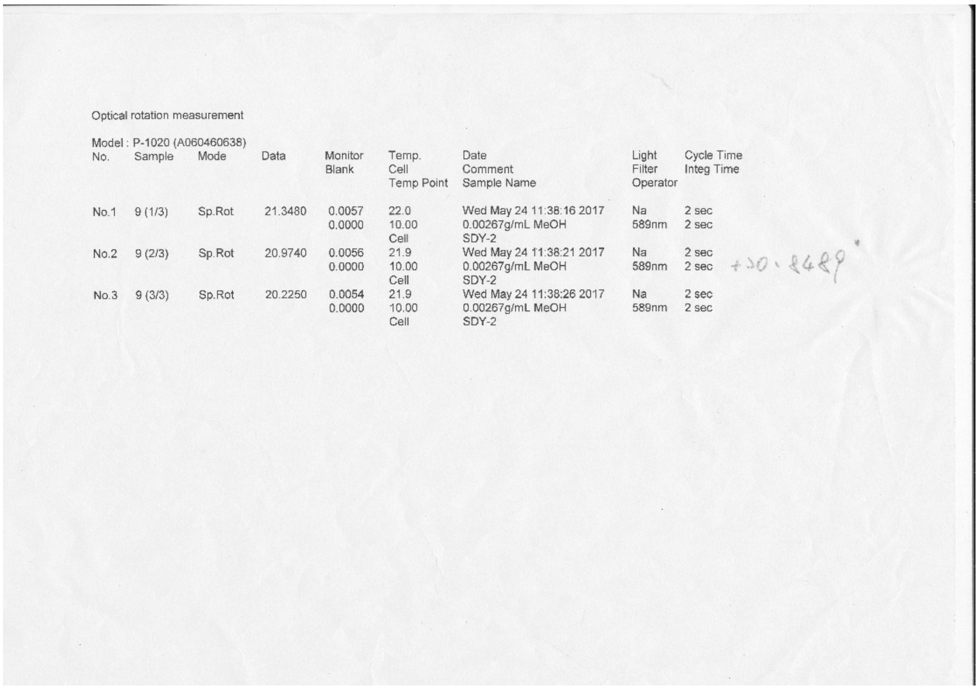

Optical rotation measurement

Model : P-1020 (A060460638)

No.	Sample	Mode	Data	Monitor<br>Blank	Temp.<br>Cell<br>Temp Point	Date<br>Comment<br>Sample Name	Light<br>Filter<br>Operator	Cycle Time<br>Integ Time
No.1	9 (1/3)	Sp.Rot	21.3480	0.0057<br>0.0000	22.0<br>10.00<br>Cell	Wed May 24 11:38:16 2017<br>0.00267g/mL MeOH<br>SDY-2	Na<br>589nm	2 sec<br>2 sec
No.2	9 (2/3)	Sp.Rot	20.9740	0.0056<br>0.0000	21.9<br>10.00<br>Cell	Wed May 24 11:38:21 2017<br>0.00267g/mL MeOH<br>SDY-2	Na<br>589nm	2 sec<br>2 sec
No.3	9 (3/3)	Sp.Rot	20.2250	0.0054<br>0.0000	21.9<br>10.00<br>Cell	Wed May 24 11:38:26 2017<br>0.00267g/mL MeOH<br>SDY-2	Na<br>589nm	2 sec<br>2 sec

Figure S18. OR report of Gypmacrophin A.

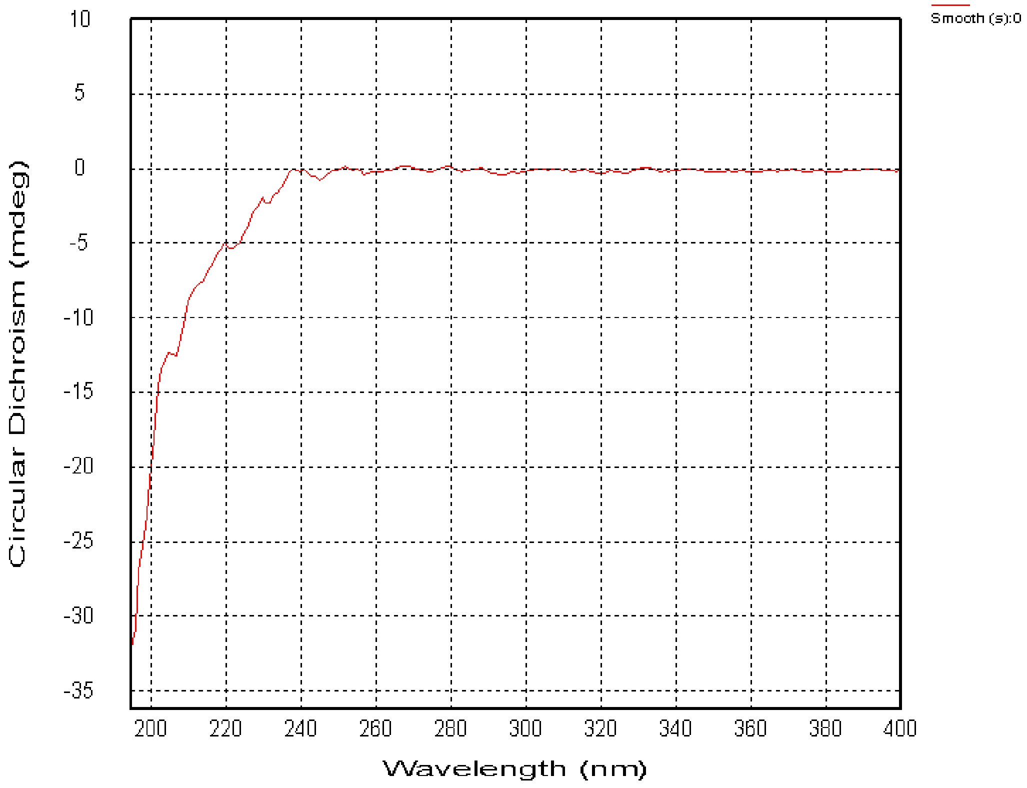

Figure S19. CD report of Gypmacrophin A.

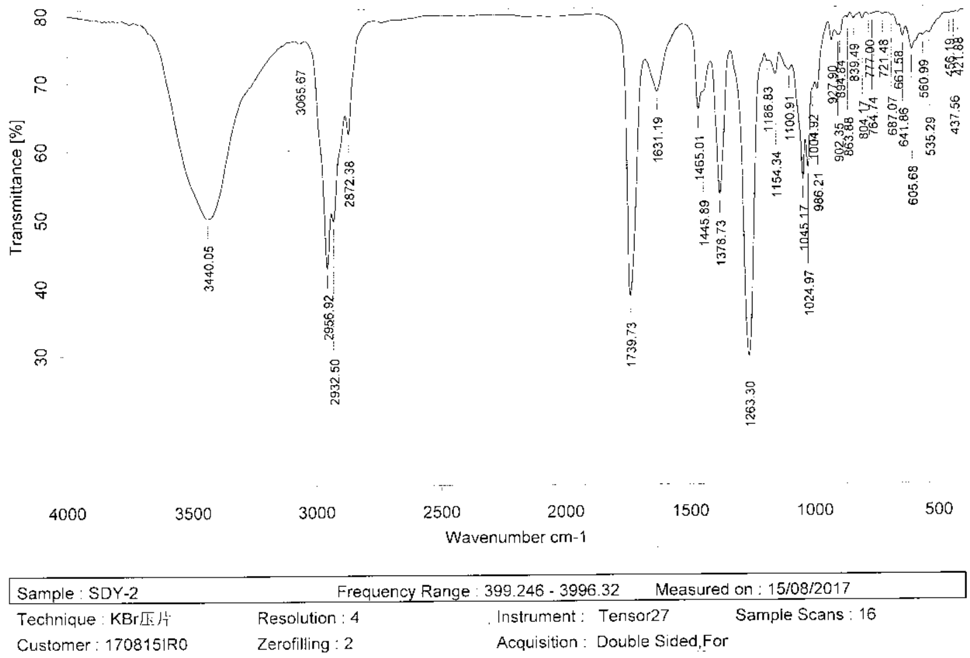

Figure S20. IR spectrum of Gypmacrophin A.

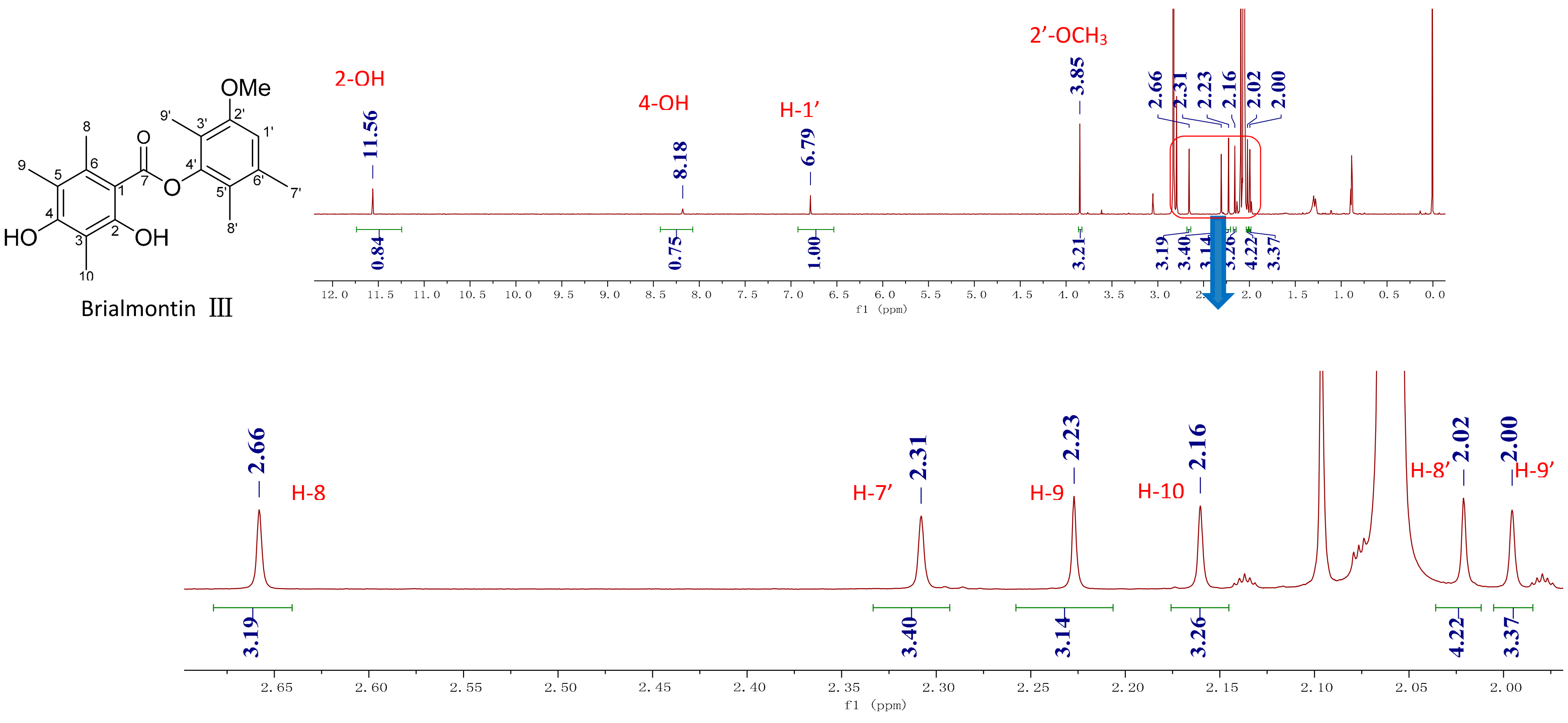

Figure S21. ^{1}H NMR spectrum of Brialmontin Ⅲ in acetone-d_6.

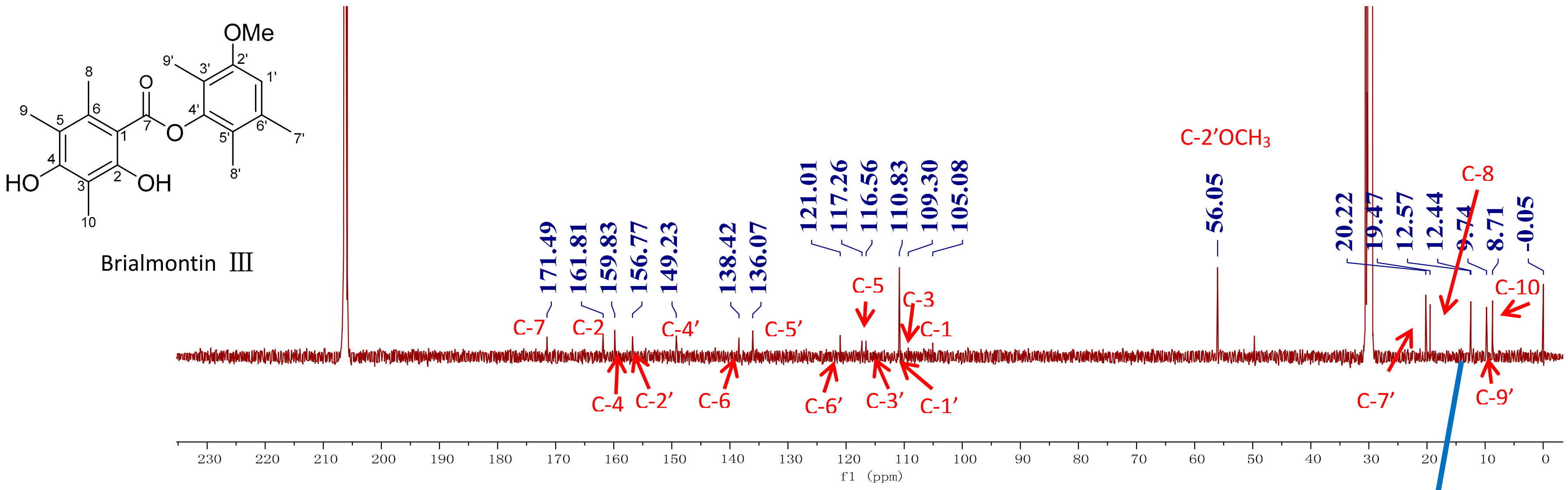

Figure S22. ^{13}C NMR spectrum of Brialmontin Ⅲ in acetone-d_6.

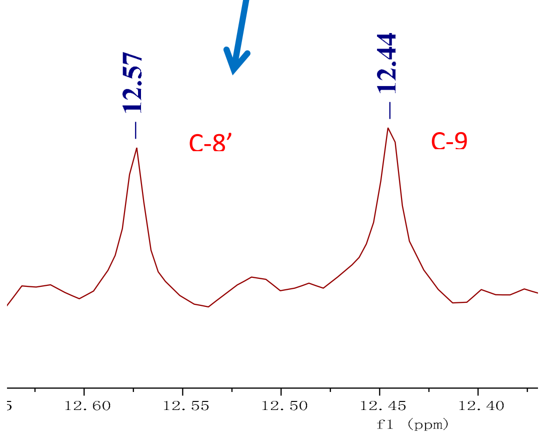

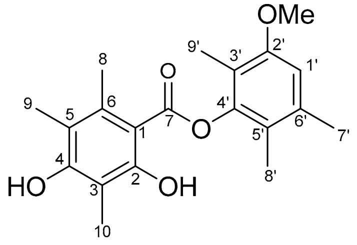

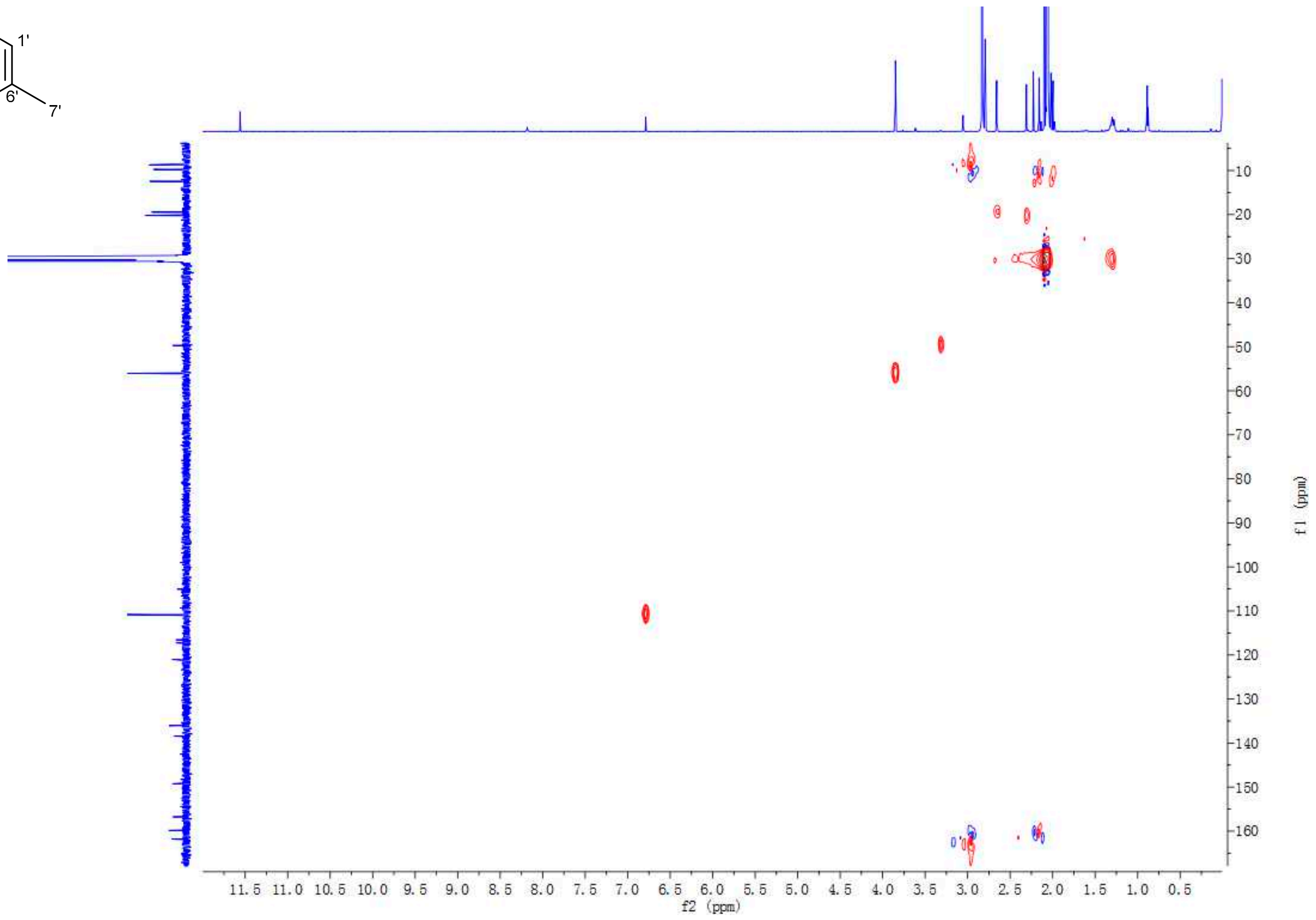

Figure S23. HSQC spectrum of Brialmontin Ⅲin acetone-*d*6.

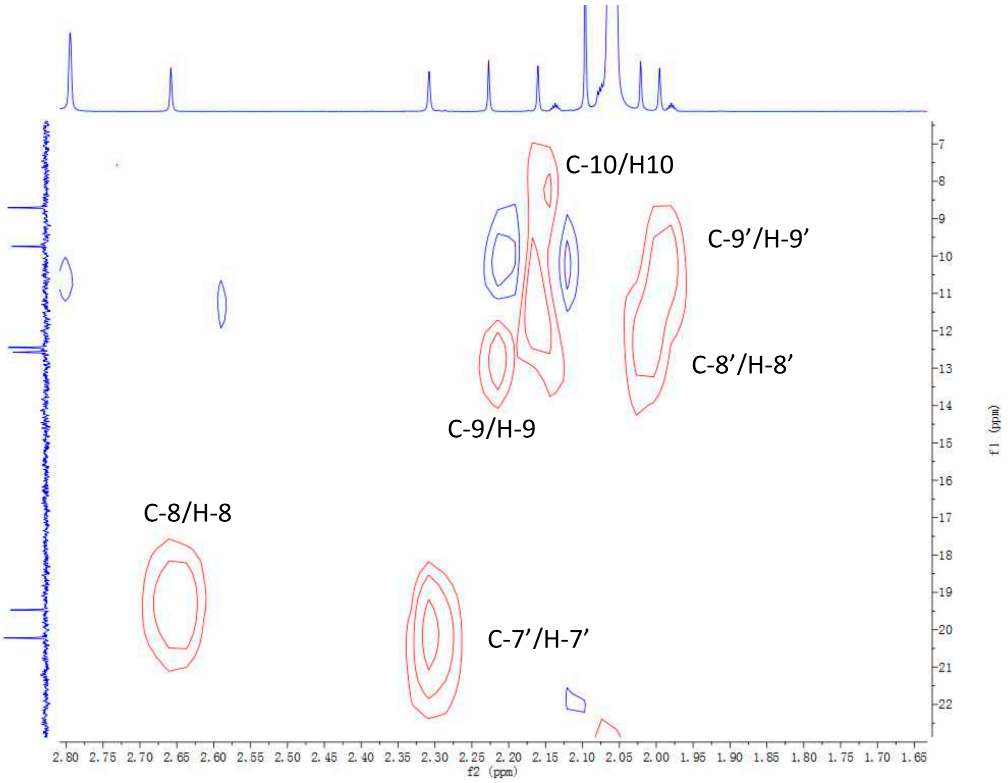

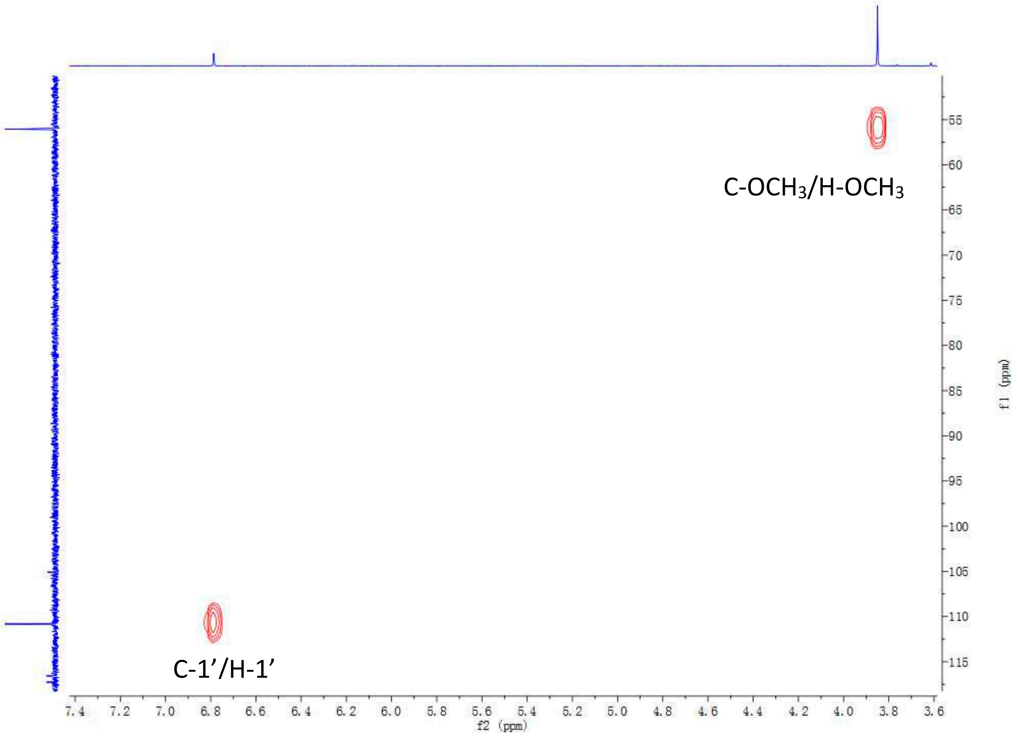

Figure S24. Expand HSQC spectrum of Brialmontin Ⅲin acetone-*d6.*

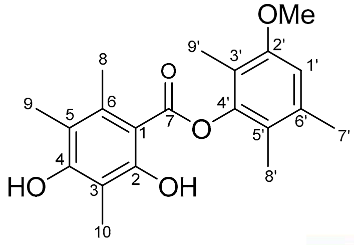

Brialmontin Ⅲ

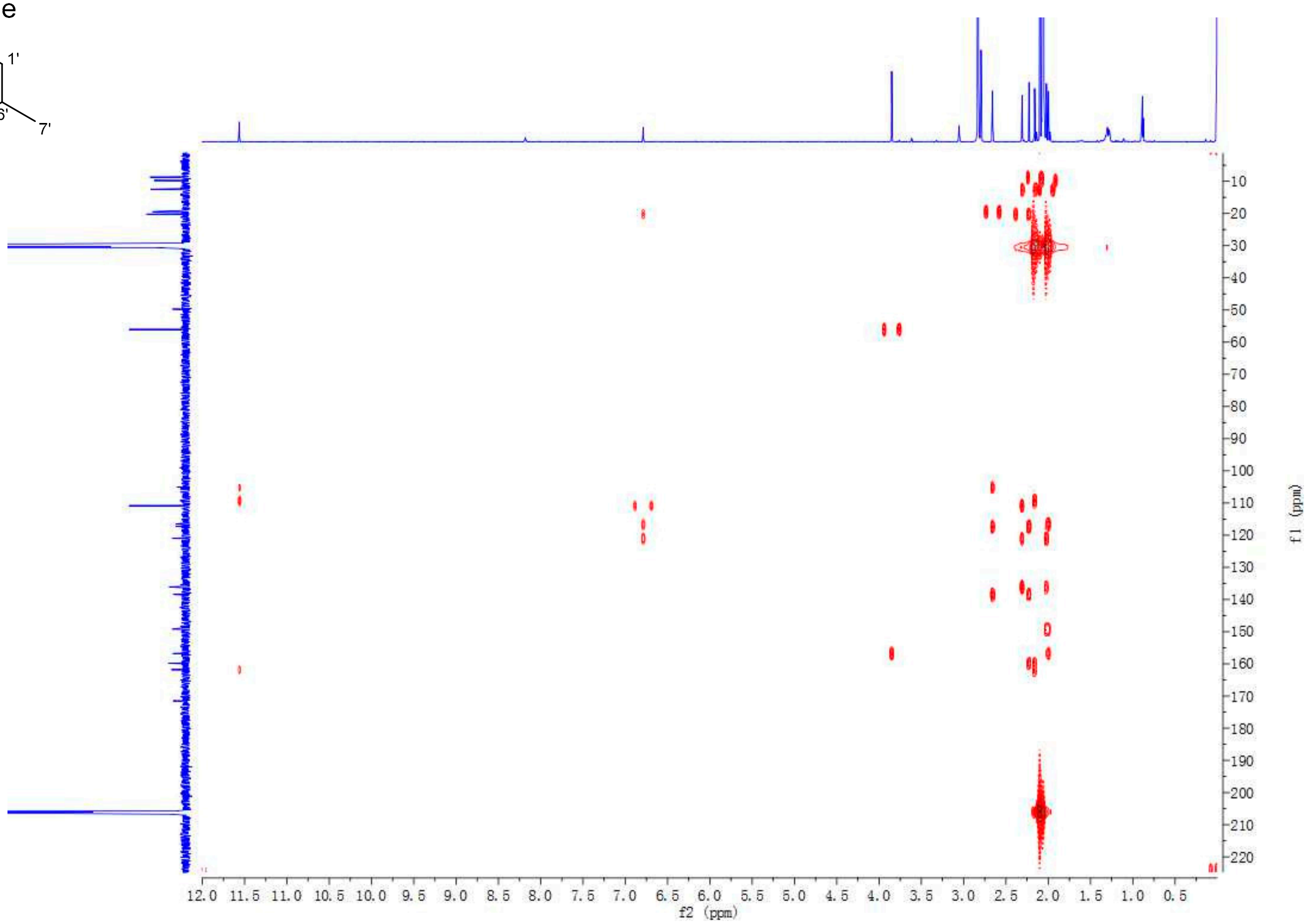

Figure S25. HMBC spectrum of Brialmontin Ⅲin acetone-*d*6.

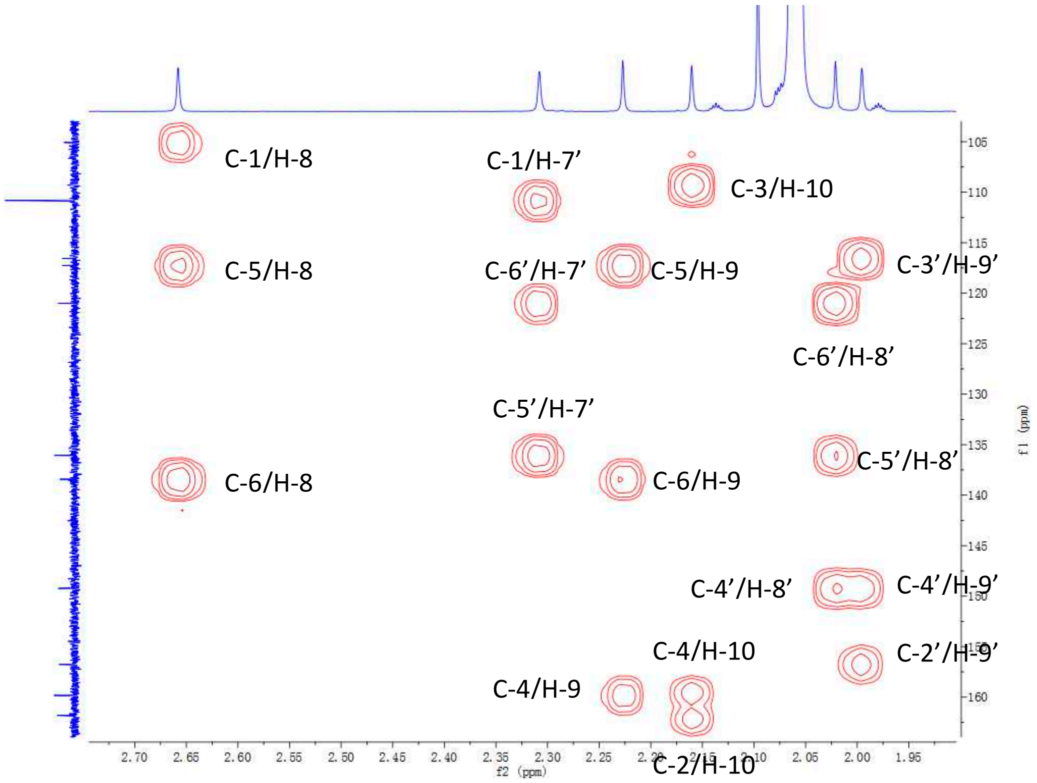

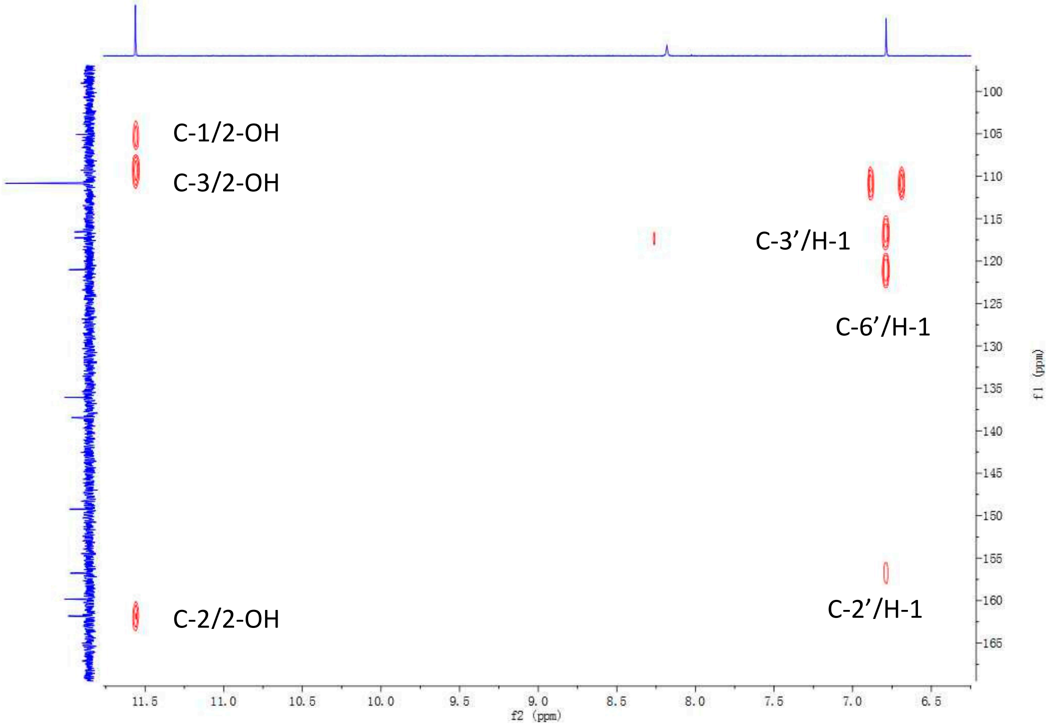

Figure S26. Expand HMBC spectrum of Brialmontin Ⅲin acetone-*d*6.

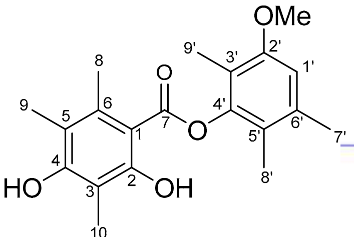

Brialmontin Ⅲ

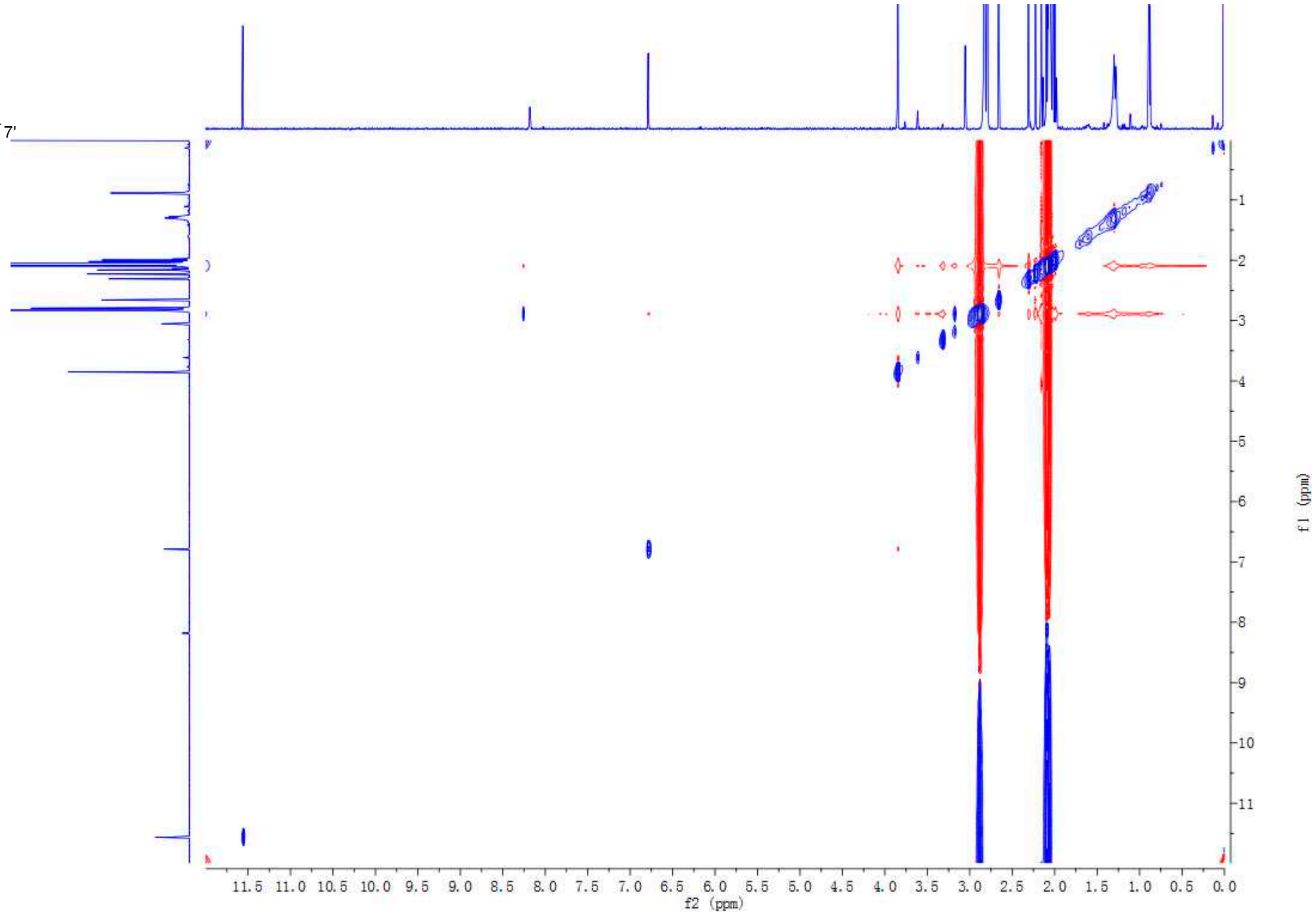

Figure S27. ROESY spectrum of Brialmontin Ⅲin acetone-*d*6.

Qualitative Analysis Report

**Data Filename**	sdy-5.d	**Sample Name**	sdy-5
**Sample Type**	Sample	**Position**	P1-E6
**Instrument Name**	Instrument 1	**User Name**	
**Acq Method**	SIBU.m	**Acquired Time**	5/16/2017 4:04:08 PM
**IRM Calibration Status**	[redacted]	**DA Method**	Default.m
**Comment**			

Sample Group **Info.**
Acquisition SW 6200 series TOF/6500 series
Version Q-TOF B.05.01 (B5125.2)

User Spectra

Fragmentor Voltage	Collision Energy	Ionization Mode
150	0	ESI

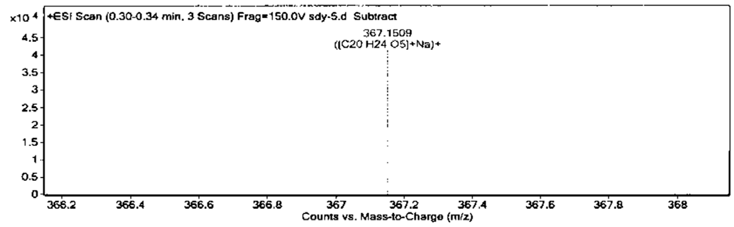

Peak List

m/z	z	Abund	Formula	Ion
179.0701	1	90849.91		
180.0734	1	9728.86		
274.2732	1	31688.52		
318.2996	1	18585.05		
367.1509	1	41454.63	C20 H24 O5	(M+Na)+
383.1246	1	42796.21		
415.21	1	12738.05		
437.1931	1	35822.31		
453.1667	1	69347.72		
454.17	1	16690.84		

Formula Calculator Element Limits

Element	Min	Max
C	3	60
H	0	120
O	0	30
N	0	5

Formula Calculator Results

Formula	CalculatedMass	CalculatedMz	Mz	Diff. (mDa)	Diff. (ppm)	DBE
C20 H24 O5	344.1624	367.1516	367.1509	1.0	2.9	9.0000

--- End Of Report ---

Figure S28.HRESIMS spectrum of Brialmontin Ⅲ.

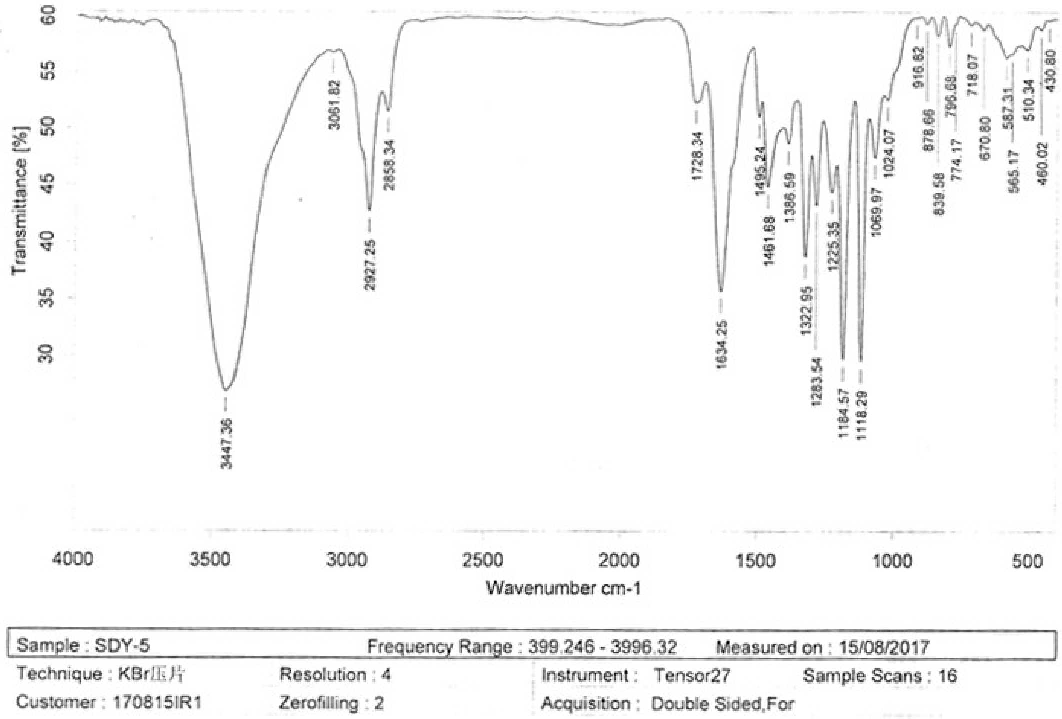

Figure S29. IR spectrum of Brialmontin Ⅲ

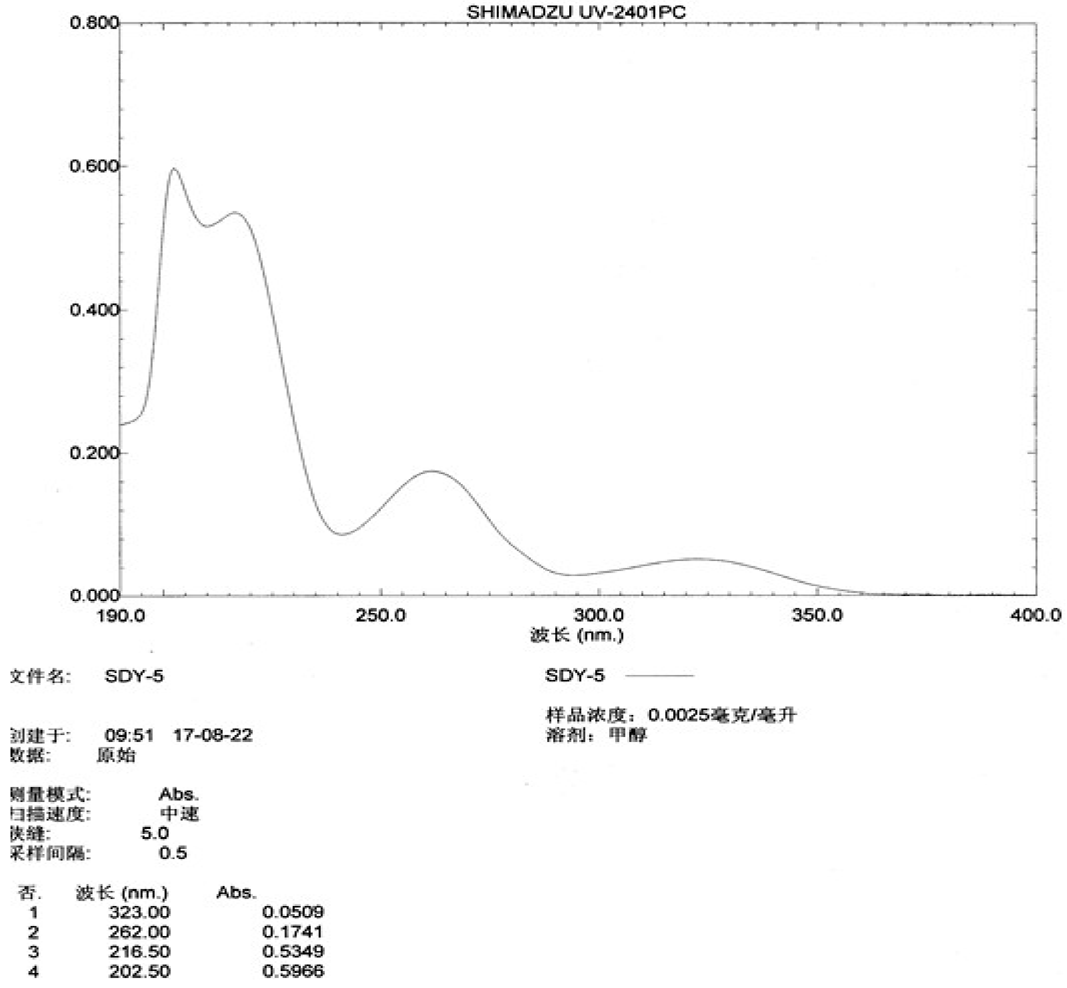

Figure S30. UV spectrum of Brialmontin Ⅲ.

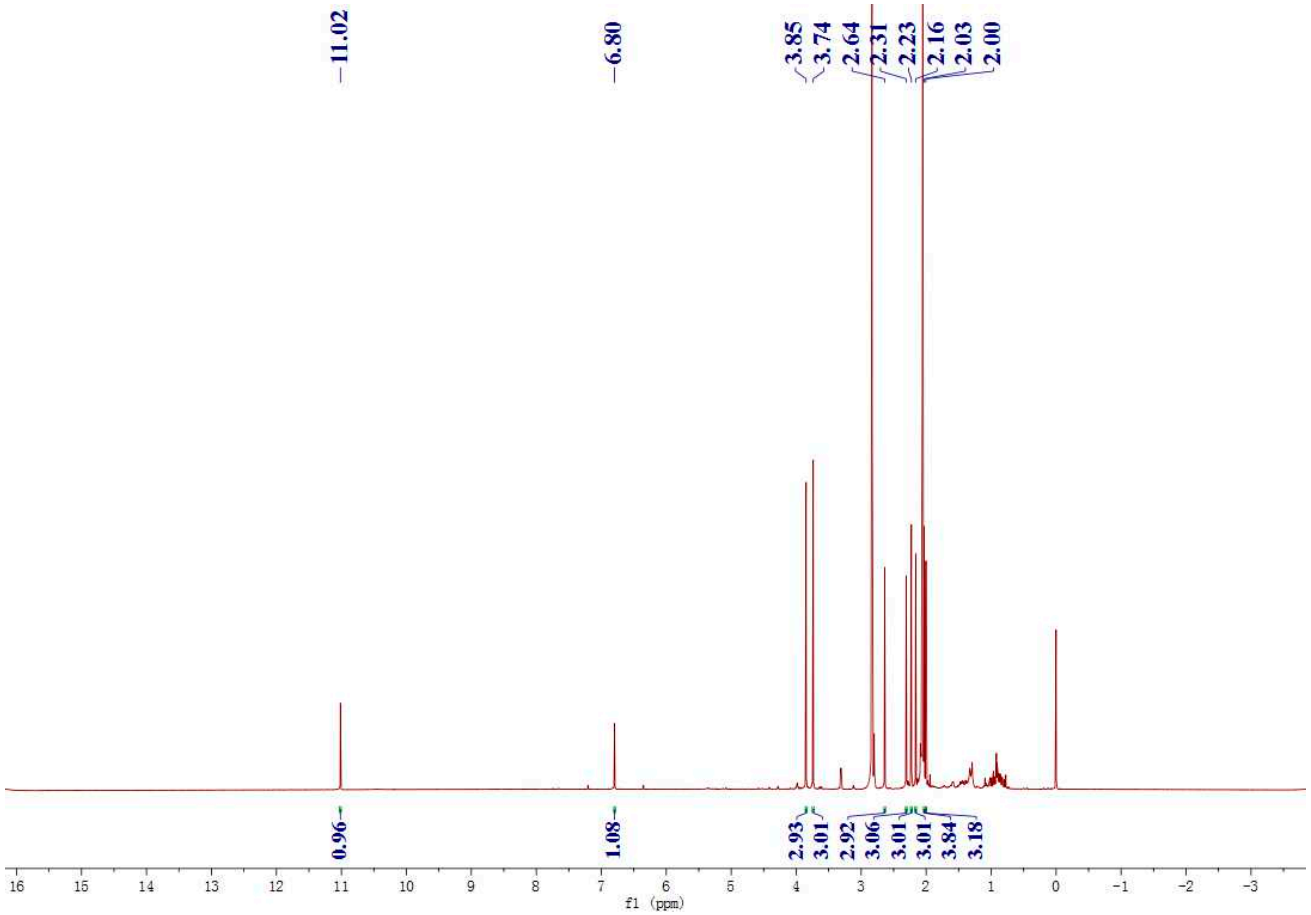

Figure S31. ^{1}H NMR spectrum of Brialmontin I in acetone-d_6.

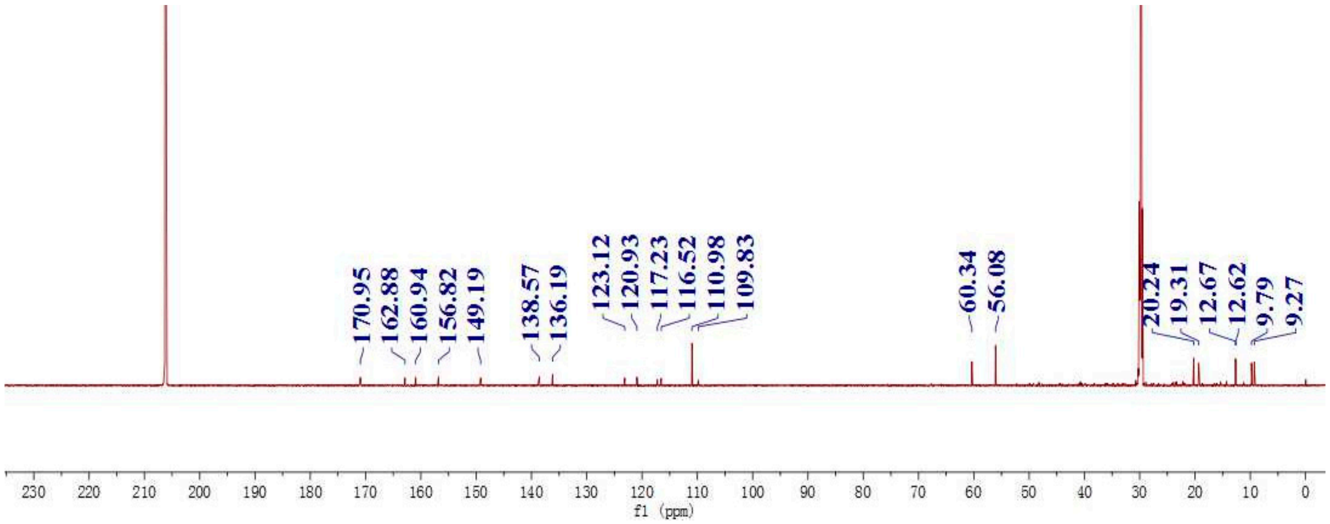

Figure S32. ^{13}C NMR spectrum of Brialmontin Ⅰ in acetone-d_6.

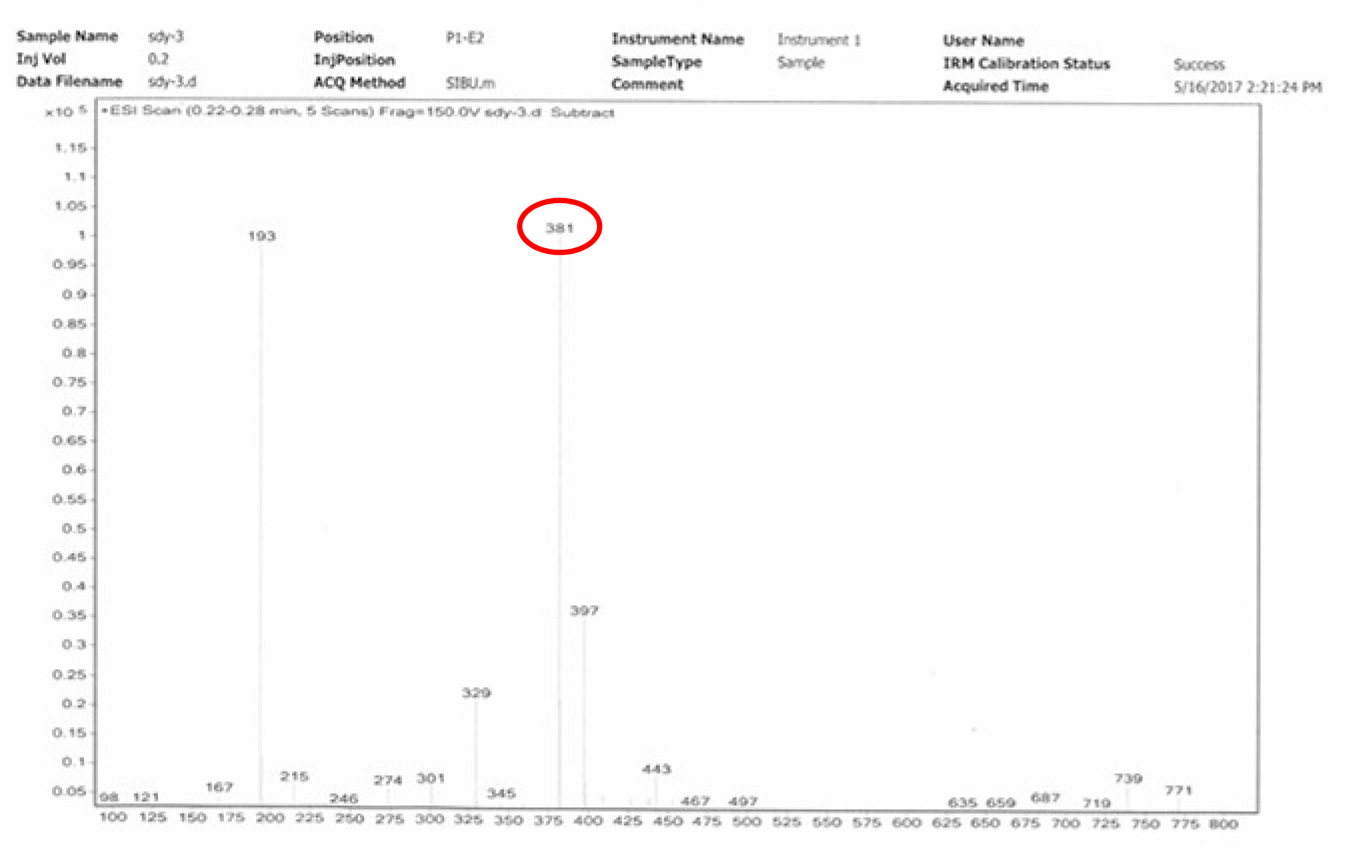

Figure S33. Positive ESI spectrum of BrialmontinⅠ.

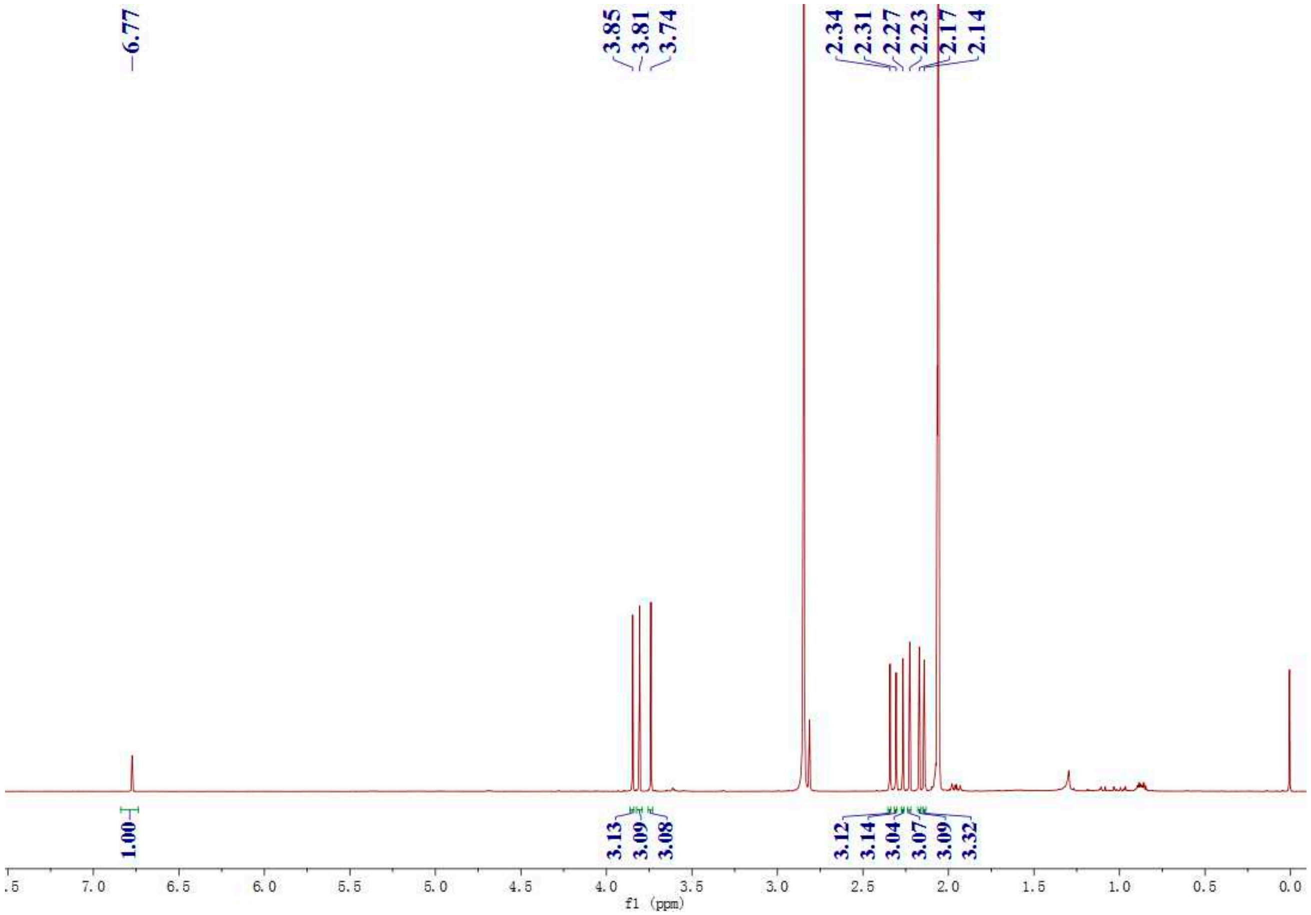

Figure S34. ^{1}H NMR spectrum of Brialmontin Ⅱ in acetone-d_6.

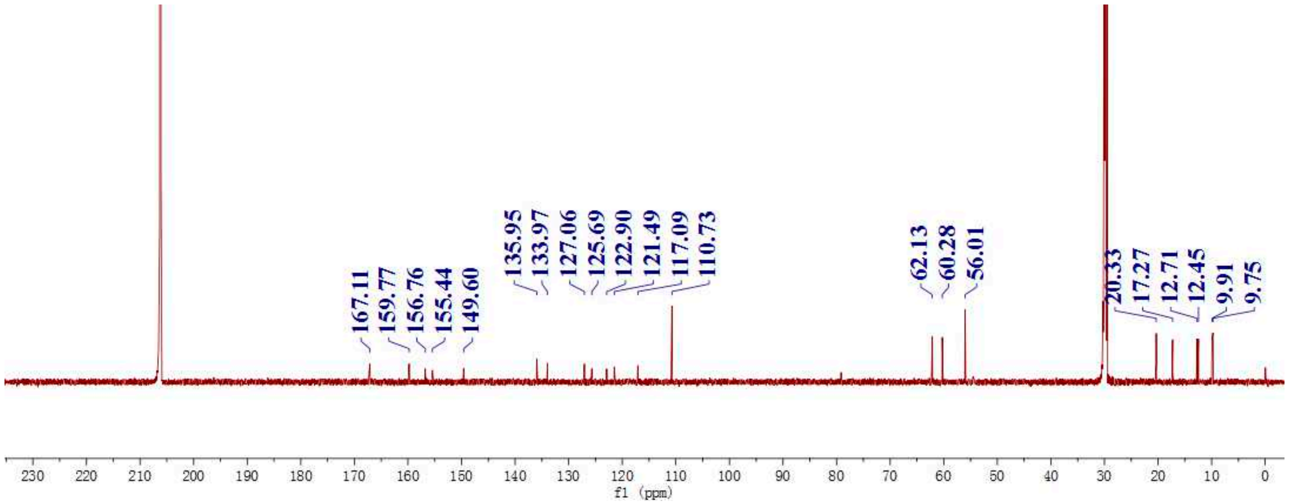

Figure S35. ^{13}C NMR spectrum of Brialmontin II in acetone-d_6.

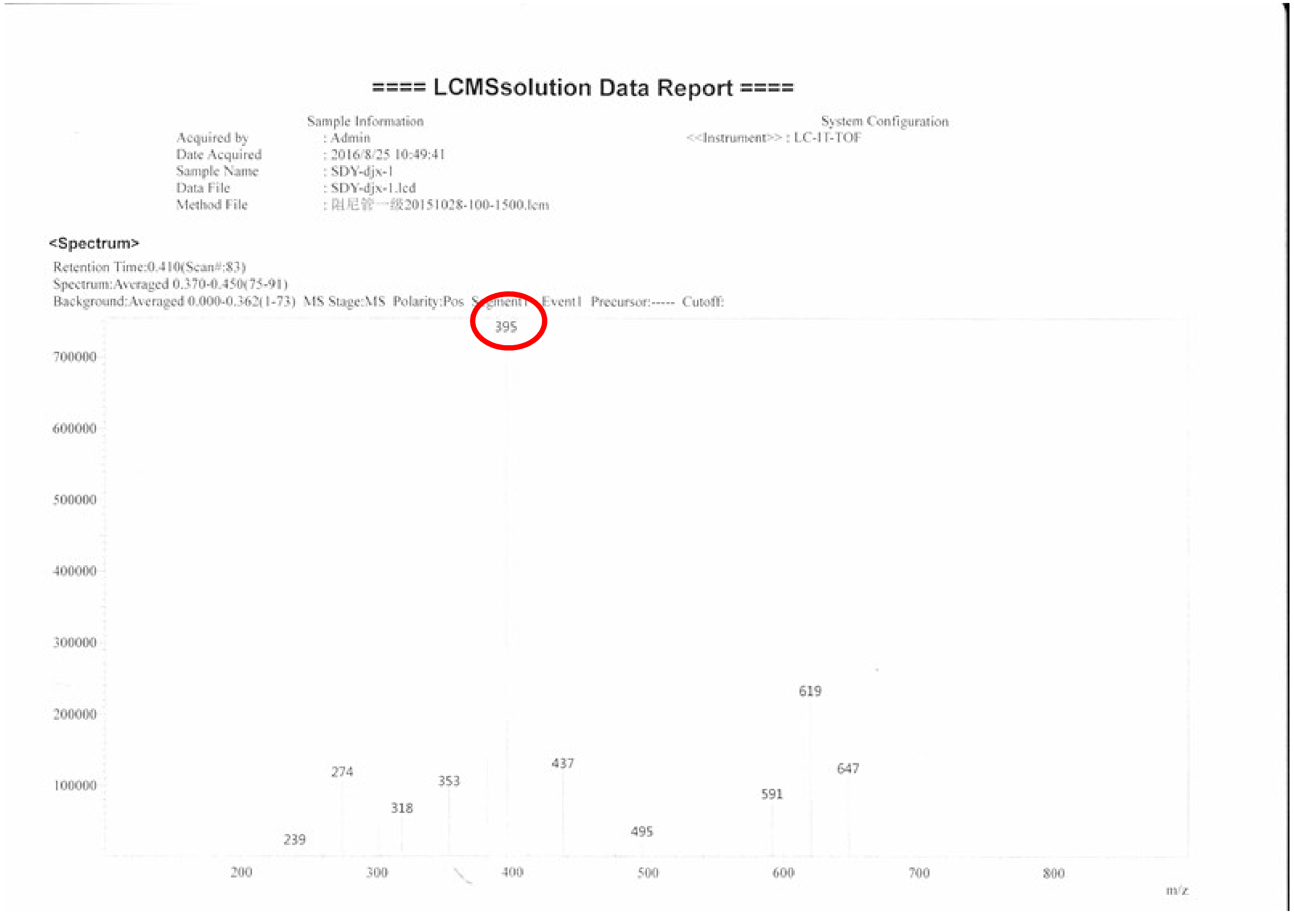

Figure S36. Positive ESI spectrum of Brialmontin Ⅱ.

No.36 Bioactive assay of Gypmacrophin A:

Acetylcholinesterase (AChE) inhibitory activity of the compounds isolated was assayed by the spectrophotometric method developed by Ellman et al [1] with slightly modification. *S*-Acetylthiocholine iodide, 5,5′-dithio-bis-(2-nitrobenzoic) acid (DTNB, Ellman's reagent), acetylcholinesterase derived from human erythrocytes were purchased from Sigma Chemical. Gypmacrophin A was dissolved in DMSO. The reaction mixture (totally 200μL) containing phosphate buffer (pH 8.0), test compound (50 μM), and acetyl cholinesterase (0.02U/mL), was incubated for 20 min (37 °C). Then, the reaction was initiated by the addition of 40 μL of solution containing DTNB (0.625mM) and acetylthiocholine iodide (0.625mM) for AChE inhibitory activity assay, respectively. The hydrolysis of acetylthiocholine was monitored at 405 nm every 30 seconds for one hour. Tacrine was used as positive control with final concentration of 0.333μM. All the reactions were performed in triplicate. The percentage inhibition was calculated as follows: % inhibition = (E - S)/E × 100 (E is the activity of the enzyme without test compound and S is the activity of enzyme with test compound). Inhibition curves were obtained for each compound by plotting the percent inhibition versus the logarithm of inhibitor concentration in the assay solution. The linear regression parameters were determined for each curve and the IC_{50} values extrapolated. The procedures are same for positive control tacrine.

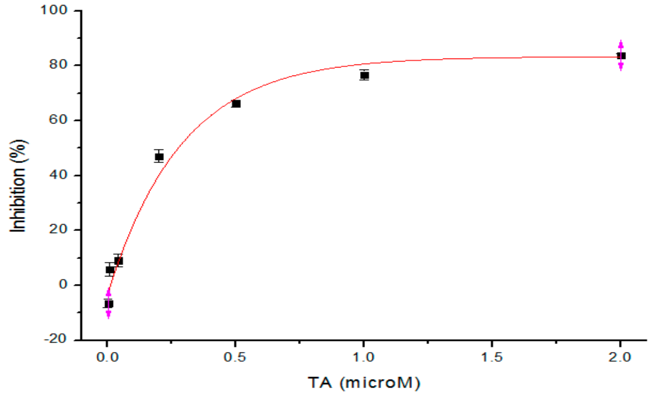

Positive control

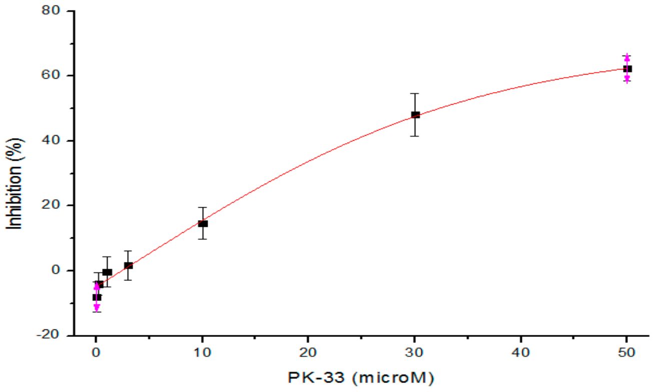

Compound 1

No. 37 ^{13}C NMR and OR calculations

The theoretical calculations of compound **1**were carried out using Gaussian 09.1[2]. Conformational analysis was initially performed using Discovery Studio 4.0 Client. The optimized conformation geometries, thermodynamic parameters, and populations of all conformations were provided in the Supporting Information. The conformers were optimized at the B3LYP/6-31G (d,p) level. Room-temperature equilibrium populations were calculated according to Boltzmann distribution law.

^{13}C NMR shielding constants of compound **1** were calculated with the GIAO method at the MPW1PW91-SCRF/6-31G (d,p) level in acetone with PCM. The shielding constants so obtained were converted into chemical shifts by referencing to TMS at 0 ppm (δcalcd= *σ*TMS– *σ*calcd), where the *σ*TMS was the shielding constant of TMS calculated at the same level. The parameters *a* and *b* of the linear regression δcalcd= *a* δexpt+ *b*; the correlation coefficient, R^2; the mean absolute error (MAE) defined as Σn |δcalcd– *δ*expt|/*n*; the corrected mean absolute error (CMAE), defined as Σn |*δ* corr– *δ* expt|/*n*, where *δ* corr= (*δ* calcd– *b*)/*a* and therefore corrects for systematic errors were presented. Rotation calculations were carried at the B3LYP/6-31+G (d,p) and B3LYP/6-311++G (2d,p) level with PCM model in MeOH based on B3LYP/6-31G (d,p) optimized geometries optical to determine absolute stereochemistry of **1**.

NO.38 Optimized conformers of 1

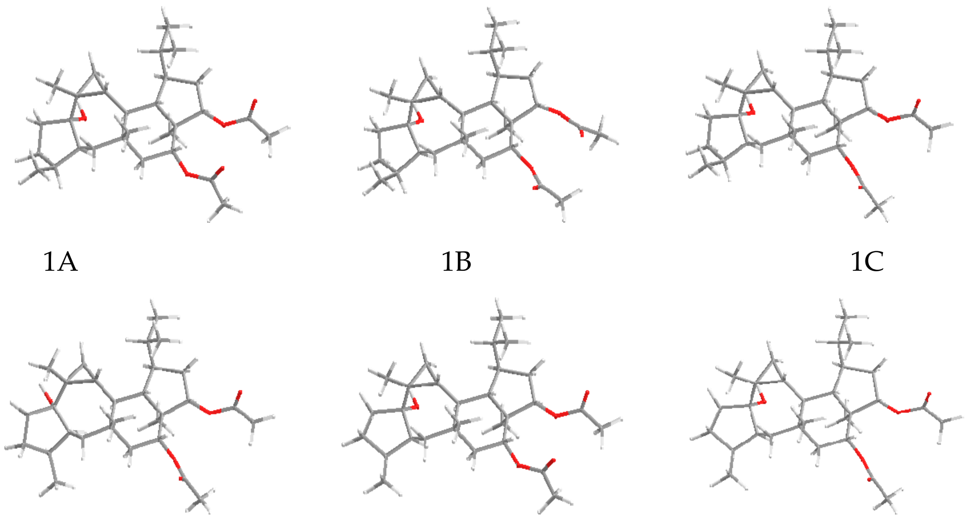

1A 1B 1C

1D 1E 1F

Figure S37. Optimized conformers of 1 (1A-1F)

Table S1. Energy analysis for conformers of **1A-1F** at B3LYP/6-31G (d,p) level in the gas phase

	E'=E+ZPE	E	H	G	P_G%
1A	-1508.194926	-1508.159508	-1508.158563	-1508.261132	39.6
1B	-1508.196014	-1508.160699	-1508.159755	-1508.261032	35.6
1C	-1508.195853	-1508.16077	-1508.159826	-1508.260025	12.3
1D	-1508.191613	-1508.155548	-1508.154604	-1508.259179	5.0
1E	-1508.193401	-1508.157923	-1508.156979	-1508.259024	4.2
1F	-1508.192333	-1508.156776	-1508.155832	-1508.258778	3.3

E, E′, H, G: total energy, total energy with zero point energy (ZPE), enthalpy, and Gibbs free energy

NO.39 Results of ^{13}C NMR calculations of 1

Table S2: Calculated ^{13}C NMR results for **1** at MPW1PW91-SCRF/6-31G (d,p) level.

NO.	1A	1B	1C	1D	1E	1F	weigh	δcal	δexp	Δ∣δcal-δexp∣	δcorr	Δ∣δcorr-δexp∣
1	155.2972	155.2672	155.4187	159.2619	157.389	157.248	155.7	40.1	40.9	0.8	39.2	1.7
2	145.8023	145.9056	145.8672	147.0736	145.3469	145.594	145.9	49.8	49.7	0.1	49.3	0.4
3	155.7733	155.6516	155.8065	157.2824	156.38665	156.4435	155.9	39.9	37.7	2.2	39.0	1.3
4	162.6961	162.6254	162.4481	165.31125	158.9975	159.0169	162.5	33.2	32.4	0.8	32.2	0.2
5	155.5058	155.5389	155.4187	155.6948	153.0049	152.969	155.3	40.4	38.8	1.6	39.6	0.8
6	111.3199	111.3249	111.0578	106.8589	110.8026	110.9169	111.0	84.7	83.3	1.4	85.0	1.7
7	168.5498	168.5388	168.3492	166.4243	167.0674	167.1655	168.3	27.4	24.2	3.2	26.3	2.1
8	179.3099	179.2409	179.225	173.7475	179.1521	179.1644	179.0	16.7	16.4	0.3	15.3	1.1
9	169.74	169.6832	169.6687	165.4313	168.9033	169.1566	169.4	26.3	24.8	1.5	25.1	0.3
10	155.7733	155.8414	155.4187	151.0968	156.38665	156.3744	155.6	40.2	38.8	1.4	39.3	0.5
11	153.0925	153.2807	153.0433	153.3578	152.0246	151.8376	153.1	42.6	39.7	2.9	41.9	2.2
12	157.0237	156.4232	157.4907	156.038	156.5384	157.1593	156.8	38.9	39.4	0.5	38.1	1.3
13	117.5621	119.6732	116.7538	117.926	119.6376	117.5651	118.3	77.4	77.3	0.1	77.6	0.3
14	144.5517	143.2726	142.9061	144.7541	143.2669	144.5444	143.8	51.9	49.4	2.5	51.4	2.0
15	150.3115	150.7645	149.6411	150.1975	150.881	150.4063	150.4	45.3	44.6	0.7	44.6	0.0
16	152.7852	152.8011	153.198	152.2698	152.7427	152.7359	152.8	42.9	42.2	0.7	42.2	0.0
17	165.5151	165.7464	165.40795	165.31125	165.7414	165.5099	165.6	30.1	28.7	1.4	29.1	0.4
18	113.5324	113.3448	116.1826	113.7364	113.3318	113.5517	113.8	81.9	81.1	0.8	82.2	1.1
19	179.6773	179.6358	179.631	178.669	178.1742	178.2313	179.5	16.2	15.9	0.3	14.8	1.1
20	170.0712	170.1022	170.1036	167.9972	171.5552	171.6254	170.1	25.6	25.8	0.2	24.4	1.4
21	173.0532	172.9449	172.9735	172.4157	172.8668	173.0194	173.0	22.8	22.8	0.0	21.5	1.3

22	184.7462	185.3258	184.9323	184.8247	185.3726	184.7552	185.0	10.7	8.7	2.0	9.1	0.4
23	165.1288	165.1212	165.40795	165.2411	165.1915	165.1801	165.2	30.6	28.4	2.2	29.5	1.1
24	172.5298	172.5411	172.5951	172.6784	172.5541	172.5201	172.5	23.2	23.2	0.0	21.9	1.3
25	180.2834	180.3546	180.5888	180.4493	180.3346	180.2709	180.4	15.4	15.5	0.1	13.9	1.6
OAc-13	29.1505	28.6046	28.6053	29.2086	28.5918	29.1555	28.9	166.9	170.4	3.5	169.4	1.0
	173.8531	173.3051	173.6432	173.8991	173.295	173.8471	173.6	22.1	21.2	0.9	20.8	0.4
OAc-18	29.0538	28.8995	28.4548	29.0549	28.8889	29.0581	28.9	166.8	170.2	3.4	169.3	0.4
	174.0098	173.9378	173.1992	174.0306	173.9453	174.0048	173.9	21.8	20.96	0.9	20.5	0.9
MAE	/	/	/	/	/	/	/	/	/	1.3	/	/
CAME	/	/	/	/	/	/	/	/	/	/	/	1.0

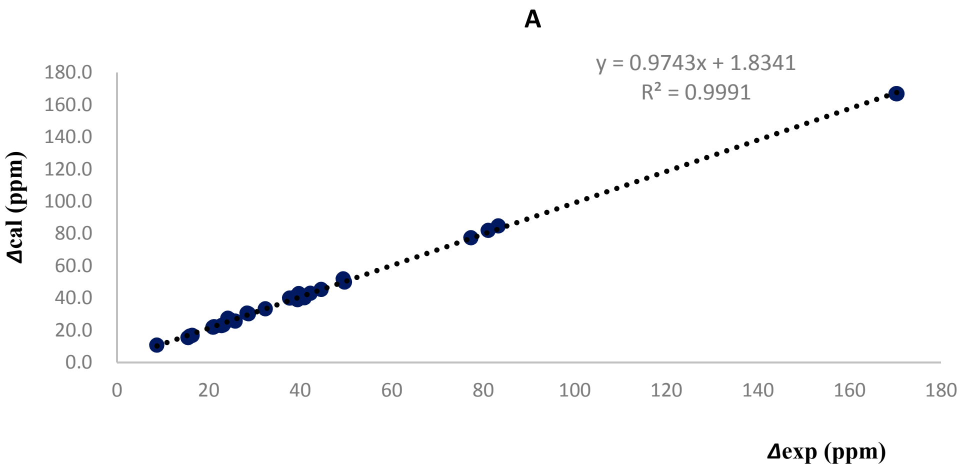

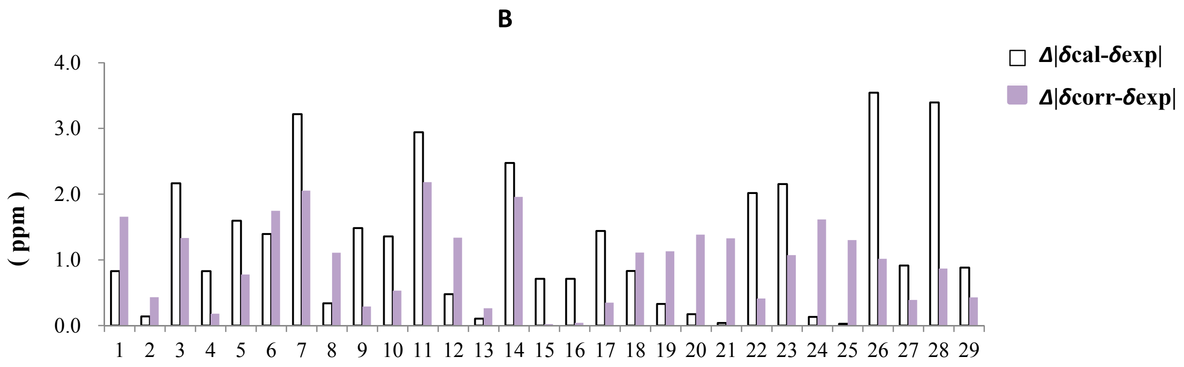

Figure S38. (A) Regression analysis of experimental versus calculated 13C-NMR chemical shifts of **1** at MPW1PW91/6-31G (d,p) level; linear fitting was shown as a line. (B) Absolute and relative chemical shift errors between δcal/δexp (MAE=1.3) and δcorr/δexp (CAME=1.0).

NO.40 Results of OR calculations of 1(2S, 3S, 6S, 7R, 9R, 10S, 11S, 13R, 14S, 15S, 16R, 18S)

Table S3. OR calculations of 1(2S, 3S, 6S, 7R, 9R, 10S, 11S, 13R, 14S, 15S, 16R, 18S) at B3LYP/6-311++G (2d,p) and B3LYP/6-31+G (d,p) level.

	E'=E+ZPE	E	H	G	P_G%	B3LYP/6-311++G (2d,p) $[\alpha]_D$	B3LYP/6-31+G (d,p) $[\alpha]_D$
1A	-1508.194926	-1508.159508	-1508.158563	-1508.261132	39.6	88.65	76.74
1B	-1508.196014	-1508.160699	-1508.159755	-1508.261032	35.6	128.98	121.84
1C	-1508.195853	-1508.16077	-1508.159826	-1508.260025	12.3	5.9	-5.9
1D	-1508.191613	-1508.155548	-1508.154604	-1508.259179	5.0	53.2	47.69
1E	-1508.193401	-1508.157923	-1508.156979	-1508.259024	4.2	132.28	130.99
1F	-1508.192333	-1508.156776	-1508.155832	-1508.258778	3.3	90.47	83.19
average	/	/	/	/	/	93.01	83.42

NO.41 Z-matrixes of **1**（1A and 1B）

Table S4. Optimized Z-matrixes of **1**（1A and 1B） in the gas phase (Å) at the B3LYP/6-31G(d) level.

C1	3.462461	-1.67393	-3.30075	C1	-4.62478	-1.84008	-0.36623
C2	4.104195	-2.79414	-2.4947	C2	-4.32264	-3.31516	-0.14447
C3	2.911723	-3.44313	-1.7926	C3	-3.24506	-3.28907	0.939171
C4	2.032805	-2.22933	-1.36146	C4	-2.33195	-2.09542	0.522602
C5	2.433576	-1.06064	-2.32761	C5	-3.23238	-1.17456	-0.3724
C6	0.538742	-2.56617	-1.24575	C6	-1.60926	-1.43578	1.706374
C7	-0.36084	-1.556	-0.45935	C7	-0.39437	-0.50831	1.371921
C8	0.066929	-0.05972	-0.69559	C8	-0.61686	0.322463	0.054355
C9	0.10672	0.245395	-2.16562	C9	-1.90509	1.090815	0.133306
C10	1.264519	-0.30569	-3.01033	C10	-3.23317	0.334904	-0.01888
C11	1.112853	1.190102	-2.76012	C11	-2.76895	1.333876	-1.07214
C12	-0.28598	-1.86851	1.065462	C12	0.894171	-1.37195	1.217394
C13	-1.0367	-0.85936	1.968068	C13	2.146775	-0.57865	0.764458
C14	-0.5952	0.594157	1.66937	C14	1.855133	0.222821	-0.52909
C15	-0.81466	0.881008	0.150483	C15	0.637559	1.163628	-0.25818
C16	-1.51742	1.681545	2.262162	C16	2.920648	1.282819	-0.88304
C17	-1.17712	2.929329	1.42388	C17	2.16605	2.252787	-1.8127
C18	-0.67018	2.418692	0.046072	C18	0.65909	2.144367	-1.45434
C19	0.870694	-0.79013	-4.39021	C19	-4.34185	0.806013	0.899136
H20	-0.88701	0.268968	-2.60115	H20	-1.84976	1.900603	0.853615
O21	3.195857	-0.13303	-1.52647	O21	-2.75105	-1.3464	-1.72252
H22	2.379467	-1.96697	-0.35116	H22	-1.55539	-2.53668	-0.11925
C23	-1.81432	-1.85116	-0.92936	C23	-0.20756	0.390237	2.628551
H24	1.083655	0.063969	-0.31692	H24	-0.71753	-0.38073	-0.77476
H25	-1.86019	0.660201	-0.0865	H25	0.866794	1.764445	0.627599
H26	-2.11066	-0.97268	1.778211	H26	2.444179	0.12731	1.550237
C27	0.863559	0.817301	2.168151	C27	1.672546	-0.74921	-1.73174
O28	-0.73594	-1.22798	3.330121	O28	3.233513	-1.49703	0.534226
C29	-1.3756	3.102292	-1.15608	C29	-0.01722	3.52442	-1.23566
C30	-1.09233	4.610182	-1.17003	C30	0.026015	4.363484	-2.51949
C31	-2.88565	2.858362	-1.24471	C31	0.540596	4.3403	-0.06492
H32	-2.5671	1.397149	2.101382	H32	3.22951	1.841187	0.011214
O33	-1.28317	1.893845	3.656545	O33	4.049979	0.76233	-1.5914
C34	-2.21923	2.64763	4.297673	C34	5.179697	0.546066	-0.86855
O35	-3.18109	3.19303	3.776315	O35	5.32244	0.764149	0.325628
C36	-1.89295	2.724975	5.757518	C36	6.247609	-0.01775	-1.75486
C37	-1.74841	-1.14796	4.233002	C37	3.909655	-1.90962	1.643613
O38	-2.89666	-0.80307	3.99543	O38	3.638333	-1.61537	2.799063
C39	-1.24943	-1.53916	5.58995	C39	5.038421	-2.8099	1.244724
C40	2.230729	-4.46732	-2.70563	C40	-3.8737	-3.19192	2.332665

H41	4.216705	-0.95353	-3.63515	H41	-5.17376	-1.69155	-1.3023
H42	3.004798	-2.11052	-4.19224	H42	-5.26393	-1.49342	0.449966
H43	4.663911	-3.48889	-3.12843	H43	-5.21455	-3.87905	0.145527
H44	4.800068	-2.37967	-1.75511	H44	-3.92581	-3.76419	-1.06311
H45	3.259929	-3.97973	-0.90125	H45	-2.66888	-4.22206	0.90435
H46	0.436435	-3.54356	-0.75472	H46	-1.24195	-2.22453	2.376994
H47	0.134029	-2.70772	-2.25527	H47	-2.34535	-0.87655	2.296557
H48	0.794814	1.816762	-3.5882	H48	-3.29188	2.283277	-1.14036
H49	1.757481	1.720624	-2.0693	H49	-2.37876	1.011182	-2.03015
H50	0.762323	-1.93117	1.38315	H50	0.713818	-2.19525	0.515369
H51	-0.68707	-2.87197	1.265771	H51	1.114483	-1.87677	2.167072
H52	-0.39938	3.531739	1.909636	H52	2.316574	1.991384	-2.86786
H53	-2.0546	3.577946	1.353225	H53	2.578133	3.259598	-1.69695
H54	0.399219	2.658432	-0.02859	H54	0.146967	1.664203	-2.29933
H55	-0.05235	-0.31392	-4.74037	H55	-4.1889	1.841833	1.223288
H56	1.649904	-0.54759	-5.11994	H56	-5.30742	0.773513	0.384234
H57	0.698018	-1.87006	-4.39562	H57	-4.39599	0.190717	1.801693
H58	3.576034	0.521057	-2.13647	H58	-3.38799	-0.90344	-2.30768
H59	-1.94116	-1.65591	-1.99924	H59	-1.08189	1.024975	2.805982
H60	-2.06283	-2.9072	-0.7663	H60	-0.07507	-0.22765	3.525346
H61	-2.57093	-1.26873	-0.39929	H61	0.664696	1.044786	2.570731
H62	1.567664	0.090848	1.754444	H62	2.59727	-1.29915	-1.94014
H63	1.249873	1.808276	1.913061	H63	0.899831	-1.50188	-1.5569
H64	0.928298	0.724693	3.258183	H64	1.400007	-0.22852	-2.65445
H65	-0.94518	2.706649	-2.07887	H65	-1.07519	3.359699	-1.01938
H66	-1.47869	5.065448	-2.08856	H66	-0.56636	5.277498	-2.40157
H67	-0.0151	4.804012	-1.13401	H67	-0.39342	3.805449	-3.36328
H68	-1.56052	5.123294	-0.32436	H68	1.046172	4.661885	-2.78008
H69	-3.30994	3.399926	-2.09785	H69	0.034094	5.310056	-0.00031
H70	-3.11235	1.799882	-1.4004	H70	0.3748	3.835038	0.890735
H71	-3.41456	3.194242	-0.34857	H71	1.61127	4.536886	-0.16793
H72	-2.64771	3.331622	6.266028	H72	5.922041	-0.97916	-2.16004
H73	-1.90151	1.722568	6.192751	H73	6.470484	0.685368	-2.56125
H74	-0.91653	3.196249	5.894655	H74	7.157947	-0.17617	-1.16961
H75	-2.07159	-1.48113	6.308976	H75	5.724023	-2.27719	0.581504
H76	-0.87881	-2.56697	5.567144	H76	5.589759	-3.11695	2.138058
H77	-0.46097	-0.8515	5.906141	H77	4.643691	-3.70263	0.753362
H78	1.449747	-5.01583	-2.17011	H78	-3.11523	-3.28867	3.115564
H79	2.956554	-5.20848	-3.05791	H79	-4.59435	-4.00292	2.484976
H80	1.775324	-4.00226	-3.58472	H80	-4.39952	-2.24511	2.486145

References:

[1] Ellman, G. L.; Courtney, K. D.; Andres jr, V.; Featherstone, R. M., A new and rapid colorimetric determination of acetylcholinesterase activity. *Biochemical Pharmacology* **1961,** 7, (2), 88-95.

[2] Frisch, M. J.; Trucks, G. W.; Schlegel, H. B.; Scuseria, G. E.; Robb, M. A.; Cheeseman, J. R.; Scalmani, G.; Barone, V.; Mennucci, B.; Petersson, G. A.; Nakatsuji, H.;Caricato, M.; Li, X.; Hratchian, H. P.; Izmaylov, A. F.; Bloino, J.; Zheng, G.; Sonnenberg, J. L.; Hada, M.; Ehara, M.; Toyota, K.; Fukuda, R.; Hasegawa, J.; Ishida,M.; Nakajima, T.; Honda, Y.; Kitao, O.; Nakai, H.; Vreven, T.; Montgomery, J. A.; Jr., Peralta, J. E.; Ogliaro, F.; Bearpark, M.; Heyd, J. J.; Brothers, E.; Kudin, K. N.;Staroverov, V. N.; Keith, T.; Kobayashi, R.; Normand, J.; Raghavachari, K.; Rendell, A.; Burant, J. C.; Iyengar, S. S.; Tomasi, J.; Cossi, M.; Rega, N.; Millam, J. M.;Klene, M.; Knox, J. E.; Cross, J. B.; Bakken, V.; Adamo, C.; Jaramillo, J.; Gomperts, R.; Stratmann, R. E.; Yazyev, O.; Austin, A. J.; Cammi, R.; Pomelli, C.;Ochterski, J. W.; Martin, R. L.; Morokuma, K.; Zakrzewski, V. G.; Voth, G. A.; Salvador, P.; Dannenberg, J. J.; Dapprich, S.; Daniels, A. D.; Farkas, O.; Foresman, J.B.; Ortiz, J. V.; Cioslowski, J.; and Fox, D. J.; Gaussian *09*, Revision B.01,Gaussian, Inc., Wallingford CT, 2010.
